# Using propensity scores to estimate the effectiveness of maternal and newborn interventions to reduce neonatal mortality in Nigeria

**DOI:** 10.1186/s12884-020-03220-3

**Published:** 2020-09-14

**Authors:** Jamie Perin, Alain K. Koffi, Henry D. Kalter, Joseph Monehin, Adeyinka Adewemimo, John Quinley, Robert E. Black

**Affiliations:** 1 Institute for International Programs, Johns Hopkins Bloomberg School of Public Health, Baltimore, MD USA; 2 Center for Child and Community Health Research, Department of Pediatrics, Johns Hopkins School of Medicine, Baltimore, MD USA; 3 USAID, Abuja, Nigeria; 4 Federal Ministry of Health, Abuja, Nigeria; 5 UNICEF, New York, NY USA

**Keywords:** Nigeria, Epidemiological transition, Effectiveness, Causal inference

## Abstract

**Background:**

Nigeria is the largest country in sub-Saharan Africa, with one of the highest neonatal mortality rates and the second highest number of neonatal deaths in the world. There is broad international consensus on which interventions can most effectively reduce neonatal mortality, however, there is little direct evidence on what interventions are effective in the Nigerian setting.

**Methods:**

We used the 2013 Nigeria Demographic and Health Survey (NDHS) and the follow-up 2014 Verbal and Social Autopsy study of neonatal deaths to estimate the association between neonatal survival and mothers’ and neonates’ receipt of 18 resources and interventions along the continuum of care with information available in the NDHS. We formed propensity scores to predict the probability of receiving the intervention or resource and then weighted the observations by the inverse of the propensity score to estimate the association with mortality. We examined all-cause mortality as well as mortality due to infectious causes and intrapartum related events.

**Results:**

Among 19,685 livebirths and 538 neonatal deaths, we achieved adequate balance for population characteristics and maternal and neonatal health care received for 10 of 18 resources and interventions, although inference for most antenatal interventions was not possible. Of ten resources and interventions that met our criteria for balance of potential confounders, only early breastfeeding was related to decreased all-cause neonatal mortality (relative risk 0.42, 95% CI 0.32–0.52, *p* <  0.001). Maternal decision making and postnatal health care reduced mortality due to infectious causes, with relative risks of 0.29 (95% CI 0.09–0.88; 0.030) and 0.46 (0.22–0.95; 0.037), respectively. Early breastfeeding and delayed bathing were related to decreased mortality due to intrapartum events, although these are not likely to be causal associations.

**Conclusion:**

Access to immediate postnatal care and women’s autonomous decision-making have been among the most effective interventions for reducing neonatal mortality in Nigeria. As neonatal mortality increases relative to overall child mortality, accessible interventions are necessary to make further progress for neonatal survival in Nigeria and other low resource settings.

## Background

Nigeria is the largest country in sub-Saharan Africa, with a neonatal mortality rate in 2016 at 36 deaths per 1000 livebirths and the second highest number of neonatal deaths in the world [1]. Although neonatal mortality in Nigeria is in decline, more progress is needed before the Sustainable Development Goal (SDG) target of 12 neonatal deaths per 1000 live births by 2030 can be met [2]. Birth asphyxia, sepsis, and complications of prematurity are the predominant causes of newborn mortality, which over time is an increasing proportion of under-five mortality in Nigeria as survival for children aged 1 to 59 months improves [1, 3]. Public health research and historic rates of newborn survival suggest that neonatal mortality can be reduced even in low resource settings [4]. Although neonates are a vulnerable population, there are cost-effective resources and interventions that promote survival without specialized technology used in high income countries for intensive neonatal care.

There is broad international consensus on which interventions can most effectively reduce neonatal mortality, including intermittent preventive treatment of malaria in pregnancy (IPTp), tetanus toxoid immunization, iron/folate supplementation [4], corticosteroids for premature births [5], active management of labor [6], early skin-to-skin contact [7], early breastfeeding initiation [8], and use of 4% chlorhexidine gel for cord care [9] or dry cord care in the absence of chlorhexidine [10]. In addition to these specific interventions for neonatal survival, it is expected that contact of both expectant mothers and neonates with local health systems will benefit neonates through the management of maternal complications and neonatal illnesses during antenatal care for pregnant women (ANC) [11], delivery in a health facility [12], skilled birth attendance (SBA) [13], and postnatal care for very young neonates (PNC) [14, 15]. There are other potentially actionable factors that may independently influence neonatal mortality such as women’s empowerment and the expected travel distance to health care [16, 17].

The national guidelines on maternal health in Nigeria recommend key interventions be provided to all pregnant women as part of antenatal and delivery care services [18]. These include tetanus toxoid immunization, iron-folate supplementation and active management of the third stage of labor. Post-partum, recommended interventions directed at improving newborn survival include early skin-to-skin contact, early initiation of breastfeeding, Vitamin K injection and chlorhexidine application to the umbilical cord. However, not all recommended interventions are widely available in all areas of Nigeria [19].

Despite recommendations from the Nigerian Federal Ministry of Health that are founded on broad-based public health studies, there are few local studies on the effects of key interventions on neonatal survival. It is possible that for health system-related or other factors, some interventions may be more or less effective than expected from primary research [20]. Recent household surveys, despite limitations, have the potential to shed light on the effectiveness of interventions for neonatal survival in Nigeria, and to identify interventions that are most effectively translated to the local context and with the greatest potential to reduce neonatal mortality. We aimed to robustly examine interventions for neonatal survival in the Nigerian setting.

## Methods

We compared neonatal mortality for those that received standard public health interventions or with health resources to those who did not based on the 2013 Nigeria Demographic and Health Survey (NDHS) and the follow-up 2014 Nigeria Verbal and Social Autopsy (VASA) Study [21, 22]. The instrument used in this study is publicly available in four languages [23]. We aimed to estimate the effectiveness of the selected interventions and resources as close as possible to what would be their causal effect. Studies using observational data to estimate effectiveness and make causal interpretations of effects often employ the probability of receiving treatment or propensity scores to adjust for potential confounders instead of regression adjustment. We used propensity scores to weight survey response so that populations with interventions or resources were more similar to those without, with respect to the covariate-predicted probability of having those interventions or resources [24]. We separately estimated the effectiveness for each of eighteen interventions and resources with potential to reduce neonatal mortality, while attempting to control for external factors, including other interventions and resources available to mothers and neonates. We aimed to estimate the effectiveness of each intervention and resource independent of other factors.

### Data

The 2013 NDHS was a multistage sample survey that used standardized methods to select households for national representation and was made publicly available for health researchers upon completion. In the first stage, census enumeration areas or clusters were selected with probability proportional to size provided by a recent population census, in several regional strata. In the second stage, complete household listings were made for the selected clusters, and households were then selected systematically with equal probability. Most interventions and resources for neonatal survival were only documented in the NDHS for the most recent birth in each household by design, so we included only the most recent household births in the five years prior to the survey [25]. The VASA surveyed households where a recent neonatal death was identified in the full birth history of the NDHS, so that additional information could be recorded. If more than one child under the age of five was indicated from the NDHS to have died in a household in the past five years, the VASA study randomly selected only one death for verbal authopsy, meaning that some neonatal deaths were not queried for cause of death. A detailed description of the methods and results from both the NDHS and VASA surveys are described in reports by the National Population Commission of Nigeria [21, 22]. Survival among neonates born in the five years prior to the 2013 NDHS was approximated using the full birth history from the women’s questionnaire. Cause of death was defined by the VASA study’s expert algorithm cause assignment [22]. For maximum consistency between information relating to those who died compared to survivors, we used the NDHS questionnaire for whether a neonate or mother received an intervention or had access to a resource. The VASA survey was used to incorporate the cause of death determined by verbal autopsy.

We selected interventions and resources for this analysis based on the Every Newborn Action Plan (ENAP) for pregnant women and neonates [26]. Some interventions recommended by ENAP were not documented in the 2013 NDHS survey, including active management of labor, corticosteroids for premature births, neonatal resuscitation, and antibiotic use for sick neonates, and so these could not be examined [27]. These interventions and resources span the continuum of care for neonatal survival, including the antenatal period, for example, whether women are primary decision maker for accessing health care, as well as whether women received antenatal care or specific antenatal interventions such as having their blood or urine tested. We chose interventions and resources to also cover the circumstances of birth, including where the neonate was delivered, whether a skilled attendant was present, and the mode of delivery. We also covered the immediate postnatal period, to include early breastfeeding, thermal care (drying, skin-to-skin contact and delayed bathing) as well as whether the neonate received postnatal care within two days of delivery. All interventions and resources included in this analysis are shown in Table 1.

**Table 1:** Description of maternal and newborn health interventions and resources expected to influence newborn survival. Estimated coverage is shown for 19,685 livebirths in the five years prior to survey, or among 12,157 livebirths occurring at home, for the most recent birth for each survey respondent in the Nigeria 2013 DHS survey

Resource/Intervention	Definition / Survey questionnaire item	Estimated Coverage (2013 Nigeria DHS)
***Resources***		
Mother is primary decision maker	Mother usually makes decisions about health care for herself.	3%
Distance is not a problem for mother’s health care	When mother is sick and wants to get medical advice or treatment, distance to the health facility is not a big problem.	69%
***Interventions delivered in the antenatal period***		
ANC 1 visit	One or more antenatal care visits during pregnancy with a skilled provider (doctor, nurse, midwife, or community health worker).	60%
ANC 4 visits	Four or more antenatal care visits during pregnancy with any provider.	51%
At least one ANC intervention	During pregnancy, mother had blood pressure measured, gave a urine or blood sample, or was told about things to look out for that might suggest problems during pregnancy.	62%
Four ANC interventions	During pregnancy, mother had blood pressure measured, gave a urine and blood sample, and also was told about things to look out for that might suggest problems during pregnancy.	37%
Tetanus Toxoid during pregnancy	During pregnancy mother was given an injection to prevent the baby from getting tetanus.	59%
Iron/folate during pregnancy	During pregnancy mother was given iron tablets or iron syrup.	63%
Malaria preventive therapy during pregnancy	During this pregnancy, mother took any drug to prevent malaria.	48%
***Interventions in labor and delivery***		
Institutional birth	Infant was delivered at hospital, health center, or health clinic.	37%
Skilled attendant during birth	Delivery was assisted by doctor, nurse, or midwife.	40%
Delivered by C-Section	Delivery was conducted by Caesarean-Section.	2%
***Interventions delivered in the postnatal period***		
Dry cord care (nothing on cord) ^a^	No substance was applied to the umbilical cord after it was cut.	64%
Neonate dried after birth ^a^	Infant dried before delivery of the placenta.	28%
Skin-to-skin contact after birth ^a^	Immediately after birth, baby was put directly on the bare skin of mother’s chest.	9%
Early breastfeeding (within one hour)	Baby was put to the breast within one hour of birth.	34%
Delayed bathing 24 h or more ^a^	Not given a bath in the first 24 h after birth.	4%
PNC within 2 days of births	Baby received care within two days of delivery from any provider (e.g. to check cord, baby’s temperature, or whether baby feeding well).	15%

^a^Only reported for home deliveries

### Potential confounding factors

In our examination of confounding, we aimed to include demographics factors that we expected would influence whether interventions or resources were available as well as neonatal survival. These factors recorded in the NDHS included birth order, whether each birth was singleton or multiple, mother’s and father’s education, maternal age at first birth, maternal age at time of index birth, whether the mother was married, urban or rural residence, household wealth quintile, and whether the surveyed household reported a prior neonatal death. We also included whether they received or had other interventions and resources as potential confounders. Some interventions were only measured in home births by the NDHS design (skin-to-skin contact, drying, dry umbilical cord treatment and delayed bathing for 24 h). We did not include these home interventions as confounders when estimating the effectiveness of interventions and resources measured both in facility and home births because of this limited population.

There were additional factors of interest likely to be related to whether an intervention or resource was available, or to the risk of neonatal mortality, that were not available due to limitations from the NDHS. For example, intrapartum complications such as preterm delivery and obstructed labor were not documented by the NHDS, and so were not available in this analysis.

### Statistical methods

We expected the differences between those who received interventions or had access to resources and those who did not to be complex [28], so we approached potential confounding carefully. Analysis using regression based methods to adjust for confounders can be subject to bias [24] and yield misleading results in circumstances with extreme confounding [29]. We aimed to estimate the effectiveness of interventions and resources while reducing bias from confounding in this population with inverse probability weights using a propensity score of the probability of receiving an intervention or resource, conditional on observed factors [30].

We used logistic regression to estimate the propensity score for each intervention or resource separately, including survey sample weights as recommended for propensity scores in complex surveys [31, 32]. We used these estimated propensity scores to weight responses with inverse probability of treatment, creating two groups, based on intervention receipt, which on average were expected to be similar in demographic factors and other interventions used to estimate the propensity score [33]. We verified this balance of potential confounders graphically after weighting with propensity scores by examining the standardized difference. The standardized difference was defined as the difference in means between treatment groups divided by the overall standard deviation. We used a cutoff for the standardized difference of 0.2 or lower to determine adequante balance of potential confounders [34].

We examined the effectiveness of interventions to prevent all cause neonatal mortality as well as for neonatal mortality due to infectious causes (sepsis, diarrhea, tetanus, pneumonia and meningitis, combined) and for mortality due to intrapartum-related events (IPRE), i.e., birth injury or asphyxia. To estimate relative all-cause, infection-specific, and IPRE-specific mortality, we used Poisson regression with propensity score weighted repsonses to estimate the effectiveness of interventions and resources [35], incorporating the survey design and the sampling probability as weights and primary sampling units as clusters [36, 37]. We used a Poisson regression model with robust variance estimation as recommended by Zou (2004) [38]. This weighted relative risk was also regression adjusted for the same factors as used in estimating the propensity score per the standard recommendation [33]. We used the product of the propensity score and the sampling weight as a composite weight in this analysis [32]. We compared this weighted estimate with an unweighted estimate that was also adjusted for demographics and other interventions in the framework of Poisson regression while incorporating the multi-stage NDHS survey design. We did not control for multiple comparisons. Data used in this analysis is publicly available for research purposes from http://www.dhsprogram.com. All analysis was conducted using the twang and survey packages in R version 3.4.0 [39]. A summary of considerations for analysis is shown in detail in Additional file 5.

## Results

There were 19,685 most recent live births by household surveyed in the five years prior to the 2013 NDHS where interventions and resources of interest were measured. Among these, estimated coverage of interventions and resources for newborn survival varied from 2% of women with caesarean delivery, to 64% of mothers who received iron or folate during their pregnancy, shown in Table 1. Additional file 1 shows the STROBE Diagram for those contributing to analysis. Demographic factors were crudely associated with neonatal mortality among surveyed births (Table 2). Singleton birth, later birth order and having no prior neonatal death in the household were strongly related to higher survival rates, while area of residence, wealth quintile, father’s education, mother’s education, mother’s marital status, mother’s age at first birth and mother’s age at index birth were not associated with newborn survival.

**Table 2:** Summary of potential confounders for maternal and newborn health interventions and resources and their associations with all-cause neonatal mortality in the Nigeria 2013 DHS survey, among deaths and survivors, for 19,685 livebirths in the five years prior to survey

		Overall	Deaths	Survivors	Crude Relative Risk	
		(*n* = 19,685)	(*n* = 538)	(*n* = 19,147)		
	Level	Percent	Percent	Percent	Est	p
Birth order	first	18%	25%	18%	(ref)	< 0.001^a^
	2nd-4th	45%	35%	45%	0.984	
	5th+	37%	40%	37%	0.991	
Multiple birth		2%	8%	2%	1.099	< 0.001
Mother’s education	None	48%	47%	48%	(ref)	0.489^b^
	Primary	19%	24%	19%	1.007	
	Secondary	33%	29%	33%	0.997	
Father’s education	None	38%	38%	38%	(ref)	0.806^b^
	Primary	18%	18%	18%	1.001	
	Secondary	41%	40%	41%	0.999	
	Missing	3%	4%	3%	1.008	
Mother’s age at first birth	< 15	8%	7%	8%	(ref)	0.030^a^
	15–17	32%	30%	32%	1.002	
	18–24	48%	47%	48%	1.002	
	25–34	12%	15%	12%	1.009	
	35+	0%	1%	0%	1.067	
Mother’s age at index birth	< 15	0%	0%	0%	(ref)	0.027^a^
	15–17	6%	8%	6%	1.009	
	18–24	31%	26%	31%	0.995	
	25–34	44%	44%	44%	0.999	
	35+	18%	22%	18%	1.004	
Mother married		92%	92%	92%	1.001	0.822
Area	Urban	36%	32%	36%	(ref)	
	Rural	64%	68%	64%	1.005	0.095
Wealth Quintile	Poorest	23%	23%	23%	(ref)	0.224^b^
	Poorer	22%	27%	22%	1.006	
	Middle	19%	16%	19%	0.996	
	Richer	18%	18%	18%	1.000	
	Richest	18%	16%	18%	0.997	
Prior neonatal death in household		16%	30%	15%	1.029	< 0.001

^a^Likelihood ratio test for association in any comparison with reference level

^b^Test for association with mortality assuming consistent trend with increasing levels

We observed considerable differences in demographic factors and especially other interventions and resources when describing those who received a specific intervention compared to those who did not. Every one of our eighteen interventions and resources had at least one potential confounder with a standardized difference above 0.2 prior to weighting with a propensity score, indicating a potential for biased associations [40]. Prior to using weights, early breastfeeding had the smallest standardized differences, where none exceeded 0.4. Facility delivery and skilled birth attendance had the largest standardized differences prior to weighting, exceeding 1.5.

**Fig. 1:**
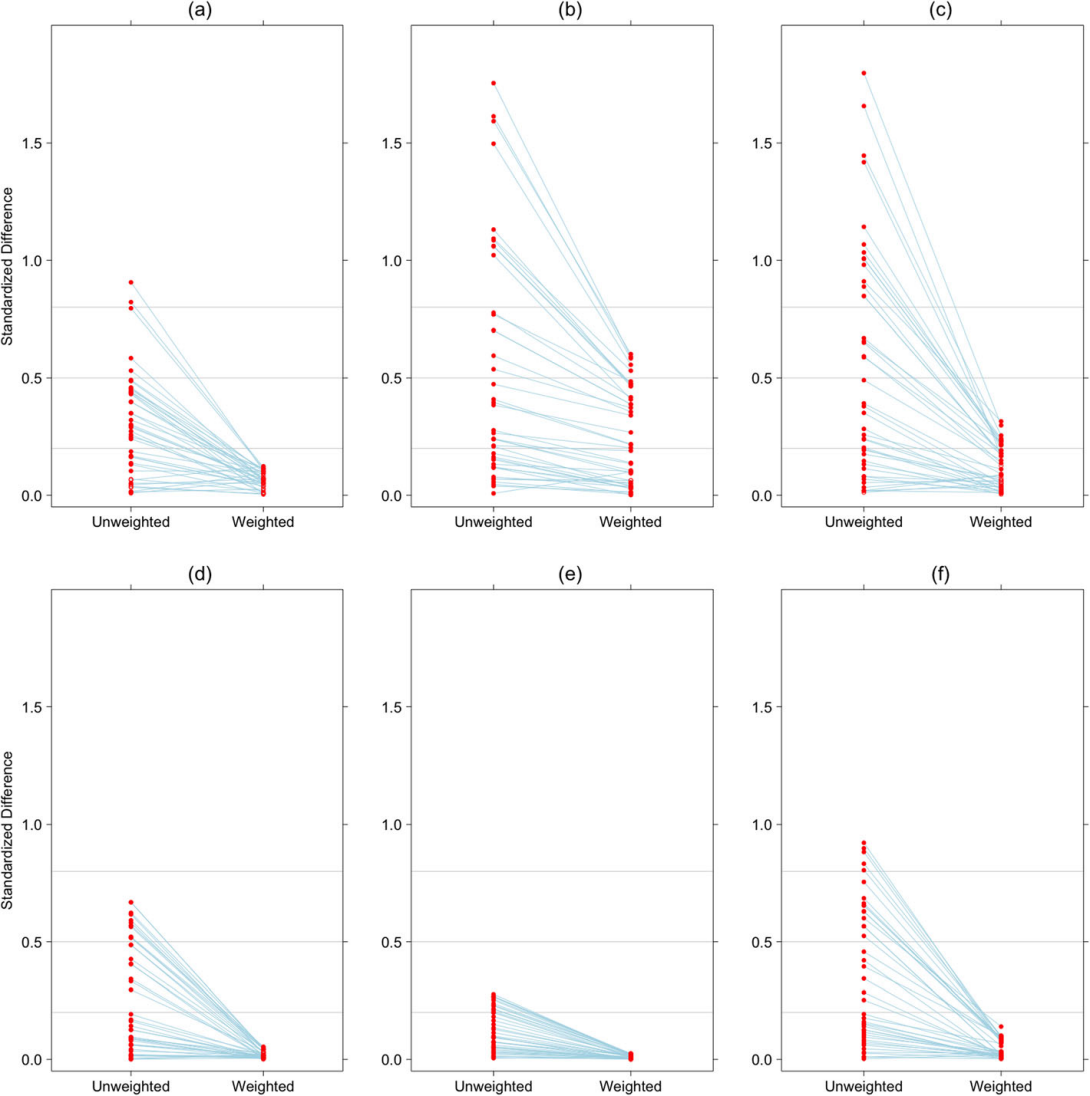
Balance in demographic and other interventions for (**a**) Mother is primary decision maker in her healthcare, (**b**) any ANC intervention received, (**c**) Skilled attendant during birth, (**d**) distance is not a problem for health care, (**e**) early breastfeeding, and (**f**) postnatal health contact within 2 days of birth. The standardized difference (average difference between those who received intervention versus those who did not divided by standard error) is shown on the vertical axis, in unweighted and propensity score weighted survey samples

Weighting surveyed births with propensity scores greatly improved the overall similarity of comparison groups. We were able to achieve optimum comparison groups for ten of eighteen interventions and resources. A summary of the balance of confounding factors between groups is shown in Fig. 1 for six interventions and resources both before and after weighting with propensity scores. Balance is shown as the average difference between those who received the intervention or resource versus those who did not divided by overall standard error (the standardized difference). Balance for all potential confounders for all 18 interventions and resources is shown in Additional file 2. Less than optimum balance of potential confounders was achieved after weighting with propensity scores for eight interventions: one ANC visit with a skilled provider (ANC1), four ANC visits with any provider, at least one ANC intervention, four ANC interventions, tetanus toxoid during pregnancy, institutional delivery, SBA, and caesarean delivery. Our ability to make unbiased inferences for these eight interventions is thus limited [40]. In general, balance was not achieved for neither demographic factors nor other interventions and resources. Unbalanced demographic factors included wealth quintile and paternal education (when comparing ANC1, tetanus toxoid, ANC4, any ANC content, four ANC interventions and facility birth), while unbalanced interventions included iron/folate receipt and tetanus toxoid vaccination during pregnancy (when comparing SBA, ANC1, ANC4, any ANC content and four ANC interventions).

The 19,685 surveyed births in the last five years included 538 neonatal deaths, although 212 (39%) of these were not covered in the VASA study and thus were not examined for underlying cause of death. Among 326 neonatal deaths with estimated cause of death, 171 (52%) were caused by sepsis, diarrhea, tetanus, pneumonia or meningitis, and 74 (23%) were due to IPRE [41]. All causes of death as well as ages at death among neonates are shown in Additional file 3.

**Fig. 2:**
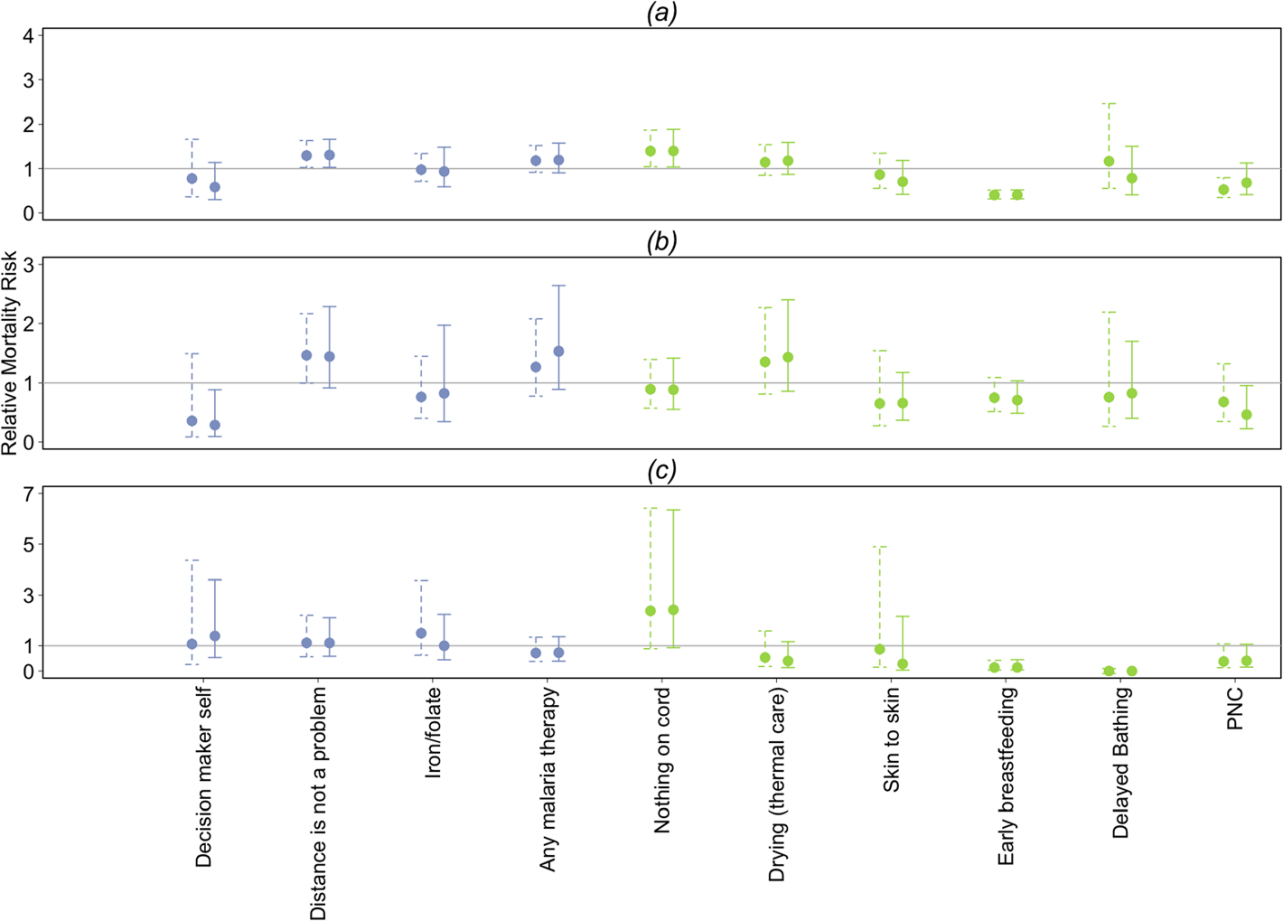
Unweighted and propensity score weighted estimated relative risk for specific interventions and resources for (**a**) all cause neonatal mortality, (**b**) neonatal mortality due to infections and (**c**) neonatal mortality due to intra-partum related events (IPRE). Both unweighted and inverse probability of treatment weighted relative risks are adjusted for demographic factor and remaining interventions, excluding those measured only in home deliveries, estimated with Poisson regression

We estimated crude associations between mortality and eighteen interventions and resources, as well as regression-adjusted associations, and associations using a propensity score for the ten interventions and resources where the standardized difference for all confounding factors was 0.2 or lower. For all-cause neonatal mortality, both unweighted and propensity score weighted relative risks are shown in Table 3 and Fig. 2 for ten interventions with adequate balance of confounding factors. Crude associations are shown in Additional file 4. There were three interventions with apparent relationships with mortality after weighting with propensity scores, although not all in the expected direction: distance not being a problem for receiving health care (relative risk 1.30, 95% CI 1.03–1.67), dry cord care (1.40, 95% CI 1.04–1.89) and early breast feeding (0.41, 95% CI 0.32–0.52). All of these in addition to PNC were also associated with all-cause neonatal mortality prior to weighting with propensity scores.

**Table 3:** Unweighted and propensity score weighted associations between MNCH interventions and all-cause neonatal mortality. Both unweighted and weighted relative risks are regression adjusted for demographic factor and remaining interventions using Poisson regression, excluding those measured only in home deliveries as indicated

Intervention	Adjusted and Unweighted			Adjusted and Weighted		
	Estimated RR	95% CI Estimated RR	p	Estimated RR	95% CI Estimated RR	p
***Resources***						
Mother is primary decision maker	0.78	(0.36, 1.66)	0.513	0.58	(0.30, 1.14)	0.114
Distance is not a problem for mother’s health care	1.29	(1.02, 1.64)	**0.031**	1.31	(1.03, 1.67)	**0.029**
***Interventions delivered in the antenatal period***						
Iron/folate during pregnancy	0.98	(0.71, 1.34)	0.881	0.94	(0.59, 1.48)	0.776
Any Malaria preventive therapy during pregnancy	1.18	(0.92, 1.52)	0.202	1.19	(0.90, 1.58)	0.213
***Interventions delivered in the postnatal period***						
Dry cord care (nothing on cord) ^a^	1.40	(1.04, 1.87)	**0.025**	1.40	(1.04, 1.89)	**0.029**
Neonate dried after birth^a^	1.14	(0.85, 1.54)	0.379	1.18	(0.87, 1.59)	0.292
Skin-to-skin contact after birth^a^	0.86	(0.55, 1.35)	0.520	0.70	(0.42, 1.18)	0.185
Early breastfeeding (within one hour)	0.40	(0.31, 0.51)	**< 0.001**	0.41	(0.32, 0.52)	**< 0.001**
Delayed bathing 24 h or more^a^	1.17	(0.55, 2.46)	0.686	0.78	(0.41, 1.50)	0.464
Postnatal health contact within 2 days of birth	0.53	(0.35, 0.80)	**0.002**	0.68	(0.41, 1.13)	0.135

^a^Only measured for home deliveries, not included in analysis for other interventions. In addition, these interventions were not adjusted for facility delivery, as they were only measured for home births

We also examined neonatal mortality due to infectious causes (Table 4) for ten interventions and resources that were balanced for potential confounders after weighting with propensity scores. Two interventions protected against mortality due to infections after weighting with propensity scores: mother is primary decision maker for her health care (relative risk 0.29, 95% CI 0.09–0.88) and PNC (0.46, 95% CI 0.22–0.95). There were no interventions having associations with mortality due to infectious causes prior to weighting with propensity scores.

**Table 4:** Unweighted and propensity score weighted associations between MNCH interventions and neonatal mortality from infectious causes (diarrhea, sepsis, meningitis, pneumonia, or tetanus). Both unweighted and inverse probability of treatment weighted relative risks are adjusted for demographic factors and remaining interventions, excluding those measured only in home deliveries marked with an asterisk, using Poisson regression

Intervention	Adjusted and Unweighted			Adjusted and Weighted		
	Estimated RR	95% CI Estimated RR	p	Estimated RR	95% CI Estimated RR	p
***Resources***						
Mother is primary decision maker	0.36	(0.09, 1.50)	0.159	0.29	(0.09, 0.88)	**0.030**
Distance is not a problem for mother’s health care	0.68	(0.46, 1.01)	0.054	0.69	(0.44, 1.09)	0.111
***Interventions delivered in the antenatal period***						
Iron/folate during pregnancy	0.76	(0.40, 1.45)	0.409	0.82	(0.34, 1.97)	0.662
Any Malaria preventive therapy during pregnancy	1.27	(0.78, 2.08)	0.343	1.53	(0.89, 2.64)	0.124
***Interventions delivered in the postnatal period***						
Dry cord care (nothing on cord) ^a^	0.89	(0.57, 1.40)	0.623	0.89	(0.55, 1.42)	0.612
Neonate dried after birth^a^	1.36	(0.81, 2.27)	0.247	1.44	(0.86, 2.40)	0.168
Skin-to-skin contact after birth^a^	0.65	(0.27, 1.55)	0.331	0.66	(0.37, 1.18)	0.159
Early breastfeeding (within one hour)	0.75	(0.51, 1.09)	0.133	0.71	(0.49, 1.03)	0.075
Delayed bathing 24 h or more^a^	0.76	(0.26, 2.19)	0.610	0.82	(0.40, 1.70)	0.599
Postnatal health contact within 2 days of birth	0.68	(0.35, 1.32)	0.256	0.46	(0.22, 0.95)	**0.037**

^a^Only measured for home deliveries, not included in analysis for other interventions. In addition, these interventions were not adjusted for facility delivery, as they were only measured for home births

For these same ten interventions, we examined neonatal mortality due to IPRE, shown in Table 5. There were two interventions with apparent relationships with mortality due to IPRE after weighting with propensity scores: early breastfeeding (0.14, 95% CI 0.04–0.45) and delayed bathing (0.00, 95% CI 0.00–0.01). All of these interventions were also associated with mortality due to IPRE in the same direction prior to weighting with propensity scores. Relative risks and their 95% confidence intervals for all-cause, infection-specific, and IPRE-specific mortality are shown in Fig. 2 for ten interventions and resources where balance of confounding factors had a standardized difference of 0.2 or lower after weighting with propensity scores.

**Table 5:** Unweighted and propensity score weighted associations between MNCH interventions and neonatal mortality due to intrapartum events. Both unweighted and inverse probability of treatment weighted relative risks are adjusted for demographic factors and remaining interventions, excluding those measured only in home deliveries marked with an asterisk, using Poisson regression

Intervention	Adjusted and Unweighted			Adjusted and Weighted		
	Estimated RR	95% CI Estimated RR	p	Estimated RR	95% CI Estimated RR	p
***Resources***						
Mother is primary decision maker	1.07	(0.26, 4.36)	0.929	1.39	(0.53, 3.60)	0.503
Distance is not a problem for mother’s health care	1.12	(0.56, 2.20)	0.754	1.12	(0.58, 2.14)	0.740
***Interventions delivered in the antenatal period***						
Iron/folate during pregnancy	1.50	(0.63, 3.58)	0.365	1.00	(0.44, 2.24)	0.996
Any Malaria preventive therapy during pregnancy	0.71	(0.38, 1.34)	0.293	0.73	(0.39, 1.36)	0.318
***Interventions delivered in the postnatal period***						
Dry cord care (nothing on cord) ^a^	2.38	(0.88, 6.42)	0.087	2.42	(0.92, 6.35)	0.073
Neonate dried after birth^a^	0.54	(0.18, 1.59)	0.260	0.40	(0.14, 1.16)	0.092
Skin-to-skin contact after birth^a^	0.86	(0.15, 4.89)	0.867	0.28	(0.04, 2.16)	0.224
Early breastfeeding (within one hour)	0.14	(0.05, 0.42)	**0.001**	0.14	(0.04, 0.45)	**0.001**
Delayed bathing 24 h or more^a^	0.00	(0.00, 0.01)	**< 0.001**	0.00	(0.00, 0.01)	**< 0.001**
Postnatal health contact within 2 days of birth	0.38	(0.13, 1.07)	0.068	0.41	(0.16, 1.06)	0.065

^a^Only measured for home deliveries, not included in analysis for other interventions. In addition, these interventions were not adjusted for facility delivery, as they were only measured for home births

## Discussion

This study aimed to describe the effectiveness of 18 interventions for neonatal survival in Nigeria using nationally representative household surveys for measuring resource and intervention coverage and cause of death estimation. Despite considerable differences in the groups that received and did not receive interventions and resources, we were able to systematically adjust for confounding factors for ten of these interventions based on a selection of demographic factors and other interventions for neonatal survival. Among the types of mortality considered (all cause, from infections, and from IPRE) we identified multiple interventions and resources with implications for neonatal survival in Nigeria. Interventions that were not identified are not necessarily ineffective or without potential effectiveness; however, these results suggest that those identified are more effective in their current implementations.

There were interventions and resources of interest that could not be examined due to extreme imbalance in the selected confounding factors, especially for antenatal care and for interventions related to labor and delivery. It is unfortunate that these interventions, which likely confer much benefit, could not be included in this analysis. This analysis suggests that there is a group of women and neonates in Nigeria that do not receive antenatal or labor and delivery care, who are very different demographically than those who do receive those resources and interventions. Further research is necessary into why such disparities exist in Nigeria and how these might be addressed.

Early breastfeeding and delayed bathing are indicated in these results for prevention of deaths due to IPRE, however, this evidence is likely due to unmeasured factors such as inability to suckle or death at a very early age relative to neonatal deaths from other causes. Of deaths attributable to IPRE, 30 (41%) out of 74 occurred on the first day on life, compared to 29 (17%) out of 171 deaths due to infection. Early breastfeeding has the strongest evidence for preventing all cause neonatal mortality, however, this result may be impacted by reverse causality, such as newborns who are born too sick to suckle due to IPRE. We repeated our anlaysis including only deaths occurring in day of life 3 or later, resulting in reduced but still notable weighted effect of early breastfeeding (relative risk 0.69, 95% CI 0.49–0.95).

Our finding also indicate that women’s decision making and postnatal care have the strongest evidence for preventing mortality due to infections. Women’s empowerment and decision-making power play a key role in the utilization of maternal, neonatal and child health care overall [42]. The severity of infections may have triggered the decision-making process as seen in other studies [43].

Our analysis also produced a counterintuitive result about dry cord care. It is possible that estimated effectiveness of dry cord care was biased through confounding by unmeasured factors. The alternatives to dry cord care in the 2013 NDHS were oil, ash, ointment/powder, animal dung, turmeric, Dettol (topical antiseptic) or methylated spirit. Chlorhexidine is now recommended for cord care by the Nigerian Federal Ministry of Health and may have been available at the time of the 2013 NDHS; however, dry cord care was recommended at the time of the survey [25]. It is also possible that this unexpected association may be due to measurement issues; it may be more difficult for women who had sick neonates or who had difficult deliveries to have known or to remember details about cord care. We also observed counterintuitive results related to distance to travel for health care not being a problem, which unexpectedly appeared to put neonates at higher mortality risk in this analysis. Other research has indicated that proximity to health care is not related to neonatal mortality [44]. We expect this result may be due to residual confounding or confounding with unmeasured factors. For example, women with complicated pregnancies may be more concerned about being far from health care.

Further details about what happens to women during their pregnancies would be a valuable addition to household surveys in analyses such as these presented here, such as if women were ill during their pregnancies or if they experienced complications during labor and delivery. Such documentation could provide valuable insight into the effectiveness of interventions, since many interventions are developed to address complications, but could also advance research on the extent of complications and the risk factors for difficult pregnancies. Future research should examine the feasibility of such reporting in household surveys. The VASA survey could serve as a model for such measurement.

Our analysis has limitations. Verbal autopsy cause assignment is not a perfect estimate for cause of death; however, verbal autopsy is likely more accurate when specifying deaths due to any infectious cause compared to death due to a specific infectious cause [41]. Given the complexities in the pathway to receiving interventions and resources, from the decision to seek care, to the receipt of care and the many factors determining quality of care, it is possible that our results are subject to residual confounding or to confounding due to unmeasured factors. Some factors of interest were available in the VASA survey, but not in the NDHS, including maternal complications during pregnancy or labor and delivery and care seeking for these, and so could not be examined for survivors. We were, however, very successful in assembling similar populations for comparison for the majority of interventions and resources of interest due to the properties of the propensity score, which has been used for estimating effectiveness of many other types of interventions [29].

We identified several interventions with the strongest evidence for promoting neonatal survival in Nigeria and similar low resource settings. Early breastfeeding, women’s autonomous decision-making and access to immediate postnatal care all have effectively reduced neonatal mortality in Nigeria. These results have important implications for where future resources can most effectively be utilized to reduce neonatal mortality in Nigeria and other similar settings. As neonatal mortality continues to be a dominant fraction of under-five mortality, accessible interventions are necessary to make further progress towards the SDGs. Selecting the most appropriate and scalable interventions will be necessary for the global health community to meet the goal of reducing neonatal mortality.

## Supplementary information

**Additional file 1.** STROBE diagram to describe those contributing to analysis.

**Additional file 2.** The standardized difference of confounders before and after propensity score weighting for 18 interventions considered.

**Additional file 3.** A summary of cause of neonatal death and the ages at neonatal death.

**Additional file 4.** Poisson regression for the association between each of 18 interventions and all cause, IPRE, and infection related mortality.

**Additional file 5.** A table summarizing the considerations of analysis at all stages.

## Abbreviations

ANC1: one or more antenatal care visits with a skilled provider
ANC4: at least four antenatal care visits with any provider
CI: confidence interval
IPRE: intrapartum-related events
IPTp: intermittent preventive treatment of malaria in pregnancy
NDHS: Nigeria Demographic and Health Survey
PNC: postnatal care for very young neonates
SBA: skilled birth attendance
SDG: Sustainable Development Goals
VASA: Verbal and Social Autopsy Study
