# Supplementary material for: Using propensity scores to estimate the effectiveness of maternal and newborn interventions to reduce neonatal mortality in Nigeria
Source: BMC Pregnancy Childbirth. 2020 Sep 14;20:534. doi: 10.1186/s12884-020-03220-3 (PMC7488987; doi:10.1186/s12884-020-03220-3)
Supplement: Supplementary file 1 — Additional file 1. STROBE diagram to describe those contributing to analysis. [file 12884_2020_3220_MOESM1_ESM.docx]

**Appendix 1. STROBE flow diagram for analytic samples.**

**Analysis of all cause mortality in association with interventions measured in all livebirths in the five years prior to survey: decision making, distance from health care, iron/folate during pregnancy, malaria preventive therapy, early breastfeeding, and postnatal health contact.**

Surveyed Live Births

Most recent by household

(N = 19,685, 538 deaths)

**Analysis of mortality due to infections or birth asphyxia in association interventions measured in all livebirths in the five years prior to survey: decision making, distance from health care, iron/folate during pregnancy, malaria preventive therapy, early breastfeeding, and postnatal health contact.**

Surveyed Live Births

Most recent by household

(N = 19,685 births, 538 deaths)

Ineligible

(N=212 deaths with no verbal autopsy)

Surveyed Live Births

Most recent by household

with cause-specific mortality

(N = 19,473 births, N=326 deaths)

**Analysis of all cause mortality in association with interventions measured only in home births: dry cord care, neonate dried after birth, skin-to-skin contact after birth, and delayed bathing 24 hours or more after birth.**

Surveyed Live Births

(N = 12,157 births, 331 deaths)

**Analysis of mortality due to infections or birth asphyxia in association with interventions measured only in home births: dry cord care, neonate dried after birth, skin-to-skin contact after birth, and delayed bathing 24 hours or more after birth.**

Surveyed Live Births

(N = 12,157 births, 331 deaths)

Surveyed Live Births and cause-specific mortality

(N = 12,037 births, N=210 deaths)

Ineligible

(N=121 deaths with no verbal autopsy)
