# Supplementary material for: Using propensity scores to estimate the effectiveness of maternal and newborn interventions to reduce neonatal mortality in Nigeria
Source: BMC Pregnancy Childbirth. 2020 Sep 14;20:534. doi: 10.1186/s12884-020-03220-3 (PMC7488987; doi:10.1186/s12884-020-03220-3)
Supplement: Supplementary file 2 — Additional file 2. The standardized difference of confounders before and after propensity score weighting for 18 interventions considered. [file 12884_2020_3220_MOESM2_ESM.pdf]

Decision maker self

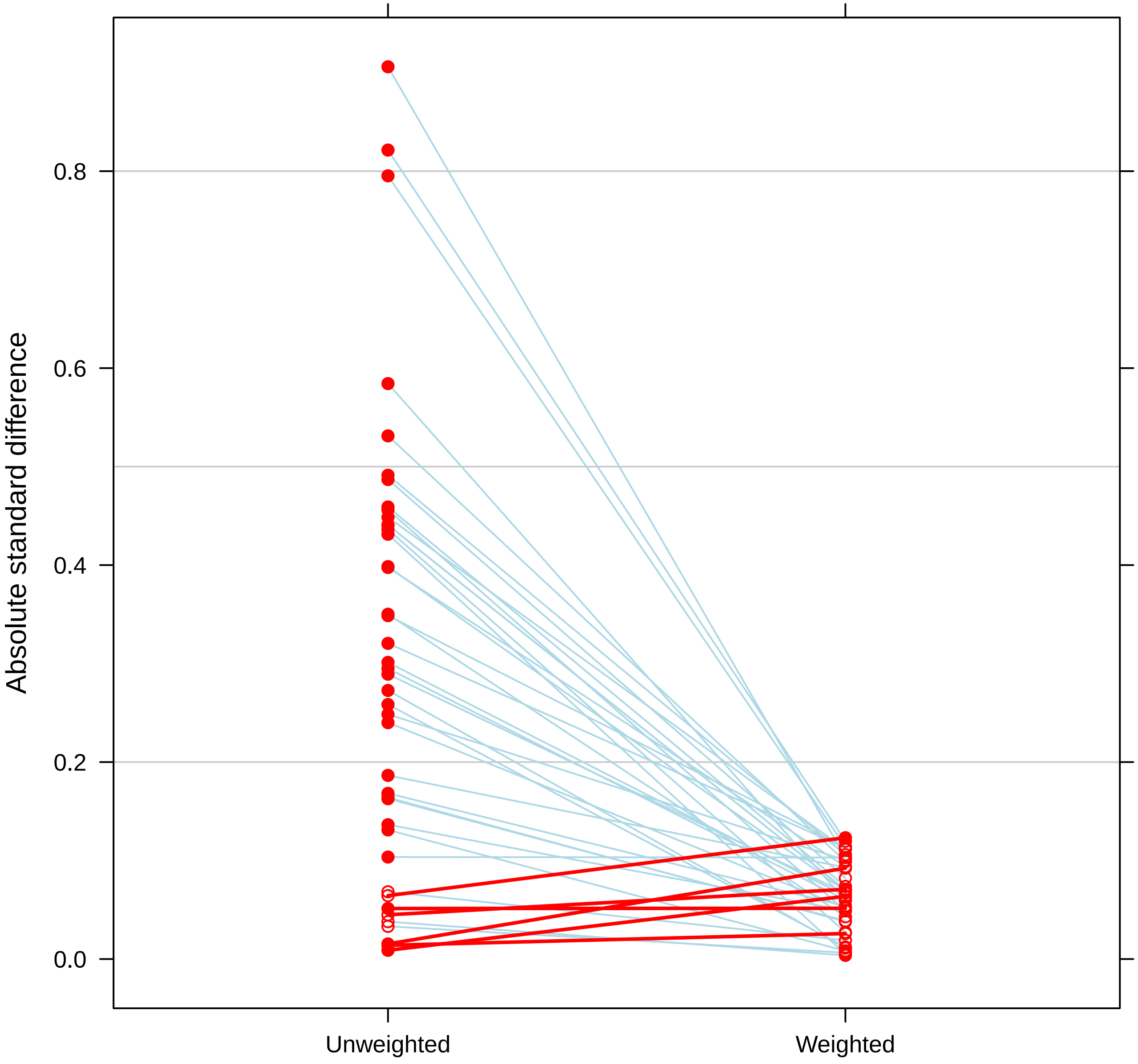

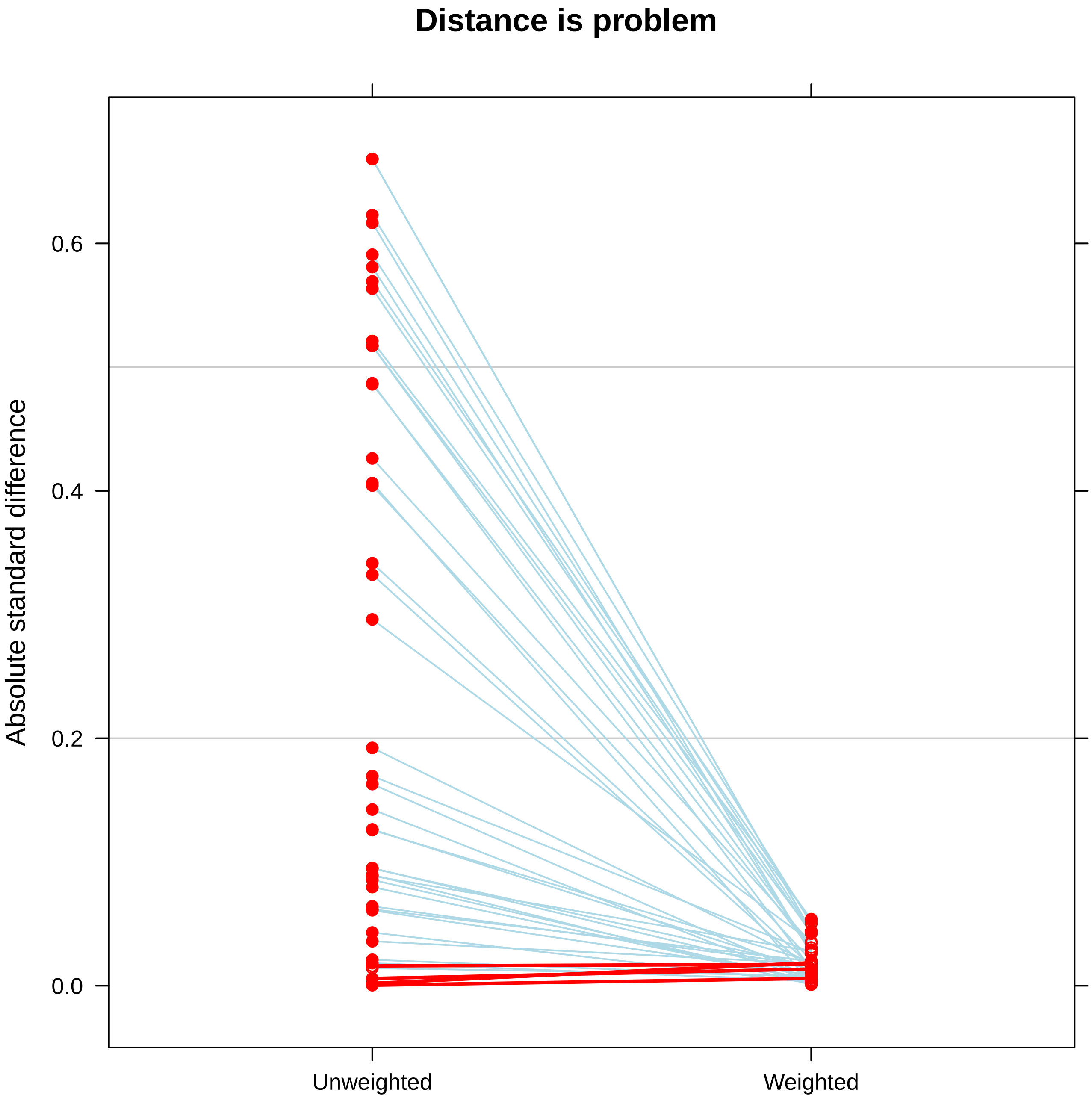

Distance is problem
Absolute standard difference
0.0
0.2
0.4
0.6
Unweighted
Weighted

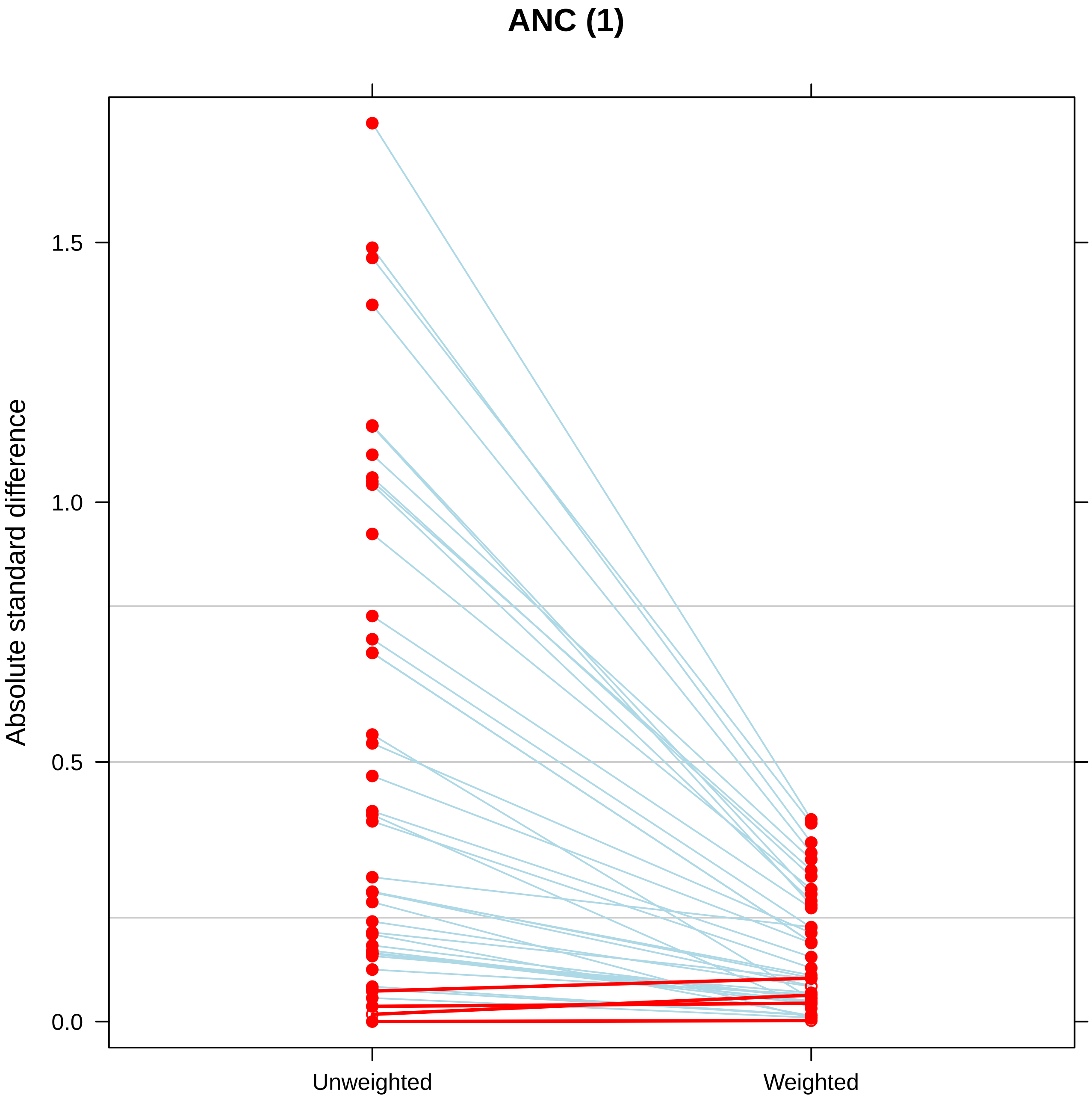
ANC (1)
Absolute standard difference
1.5
1.0
0.5
0.0
Unweighted
Weighted

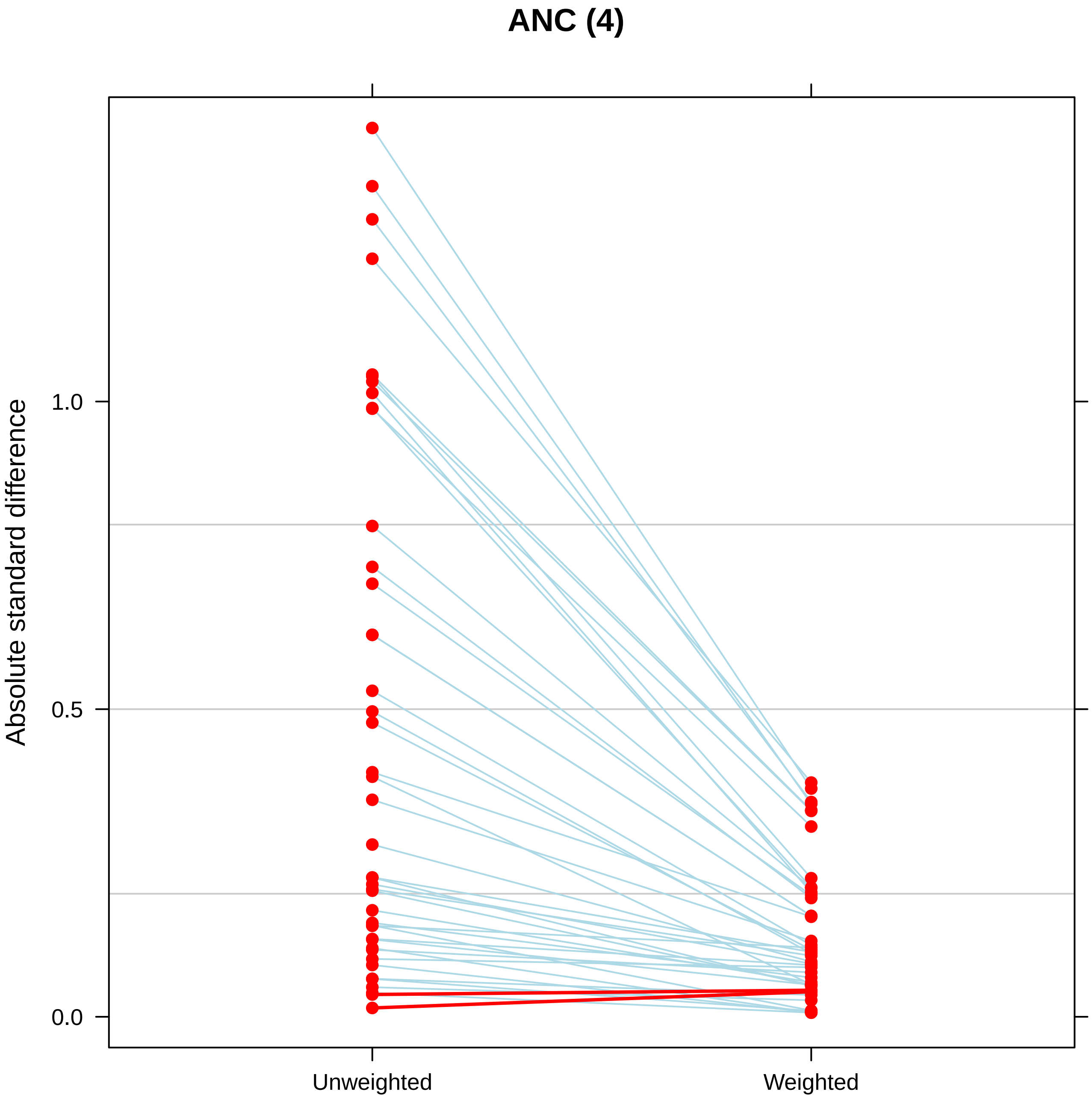

ANC (4)
Absolute standard difference
1.0
0.5
0.0
Unweighted
Weighted

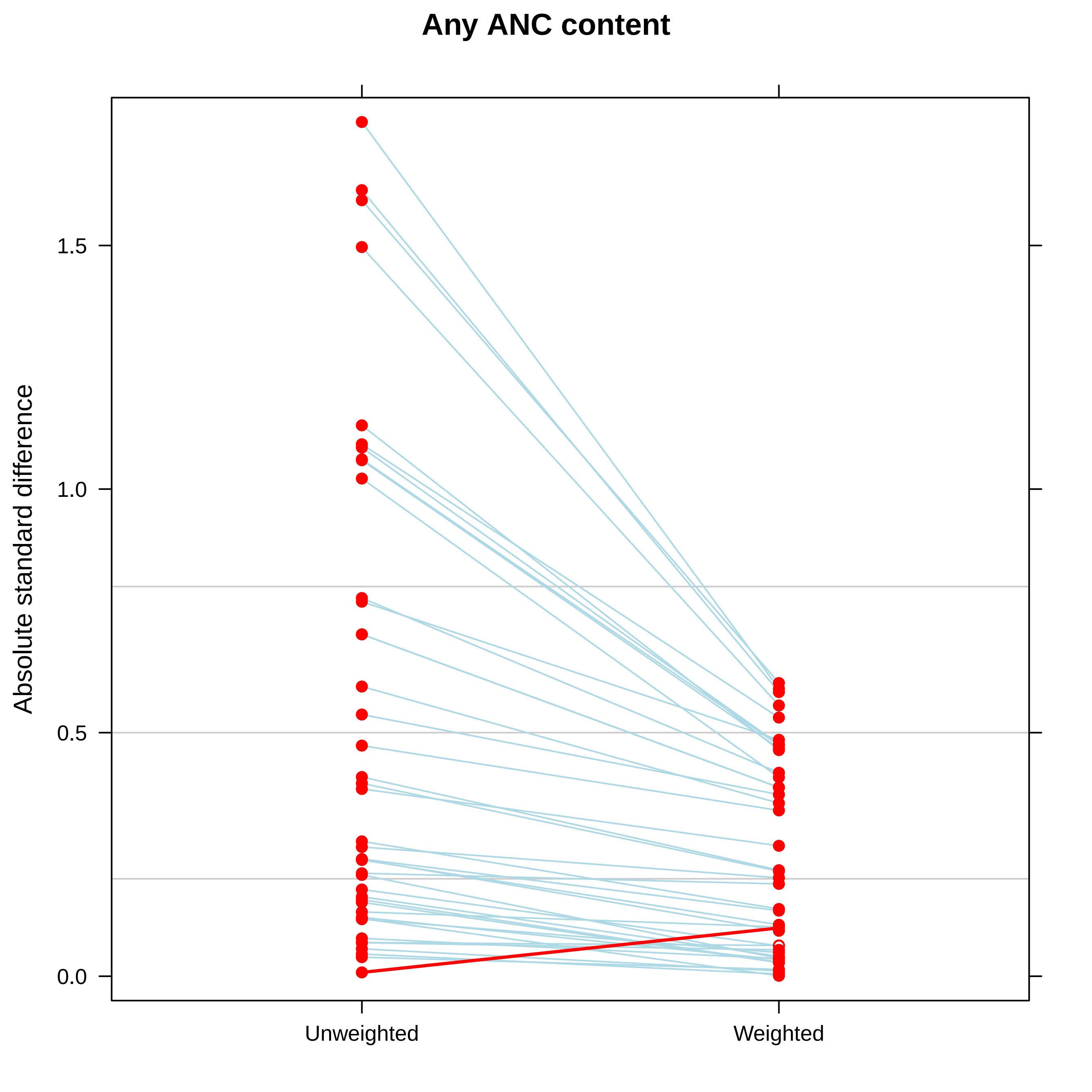

Any ANC content
Absolute standard difference
0.0
0.5
1.0
1.5
Unweighted
Weighted

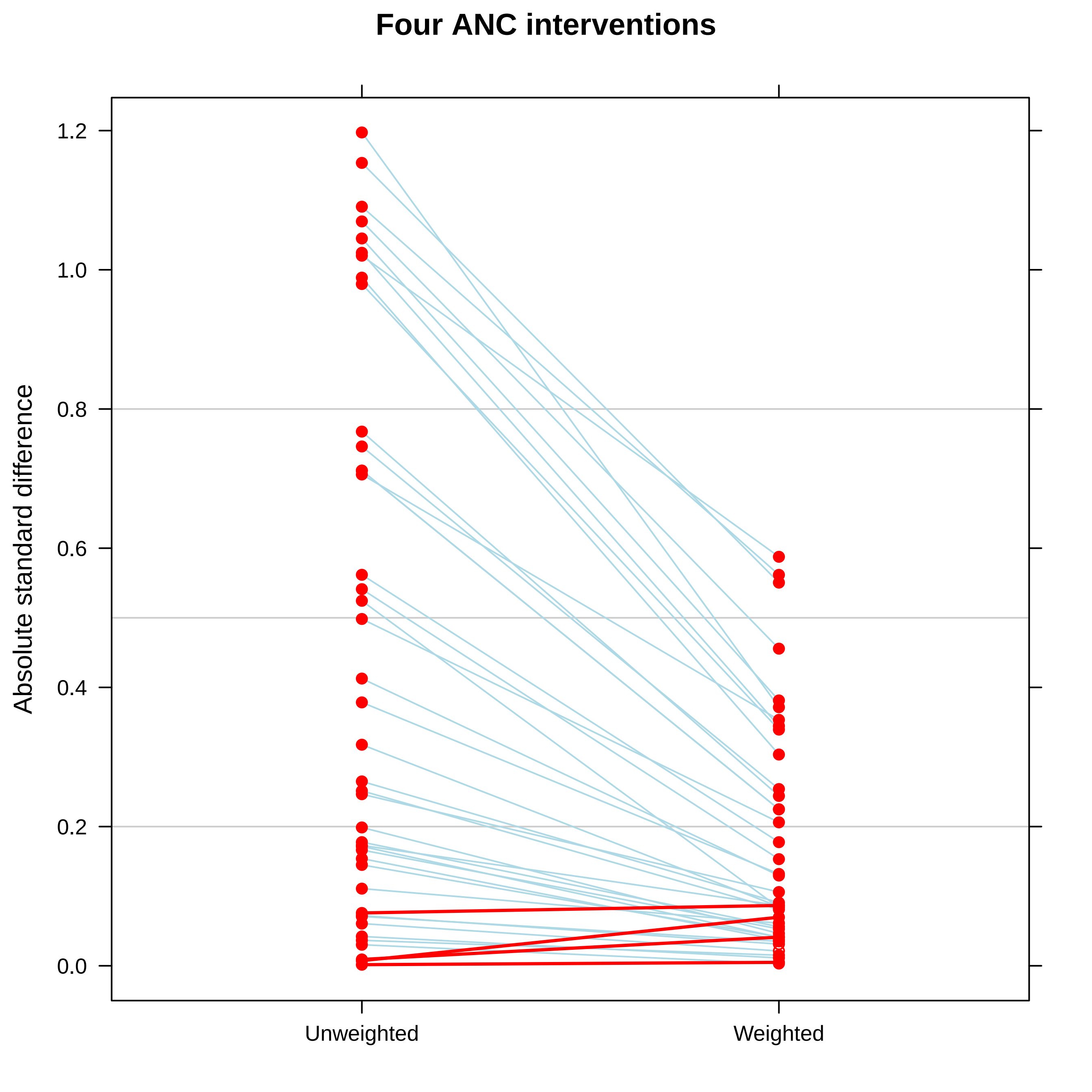

Four ANC interventions
Absolute standard difference
0.0
0.2
0.4
0.6
0.8
1.0
1.2
Unweighted
Weighted

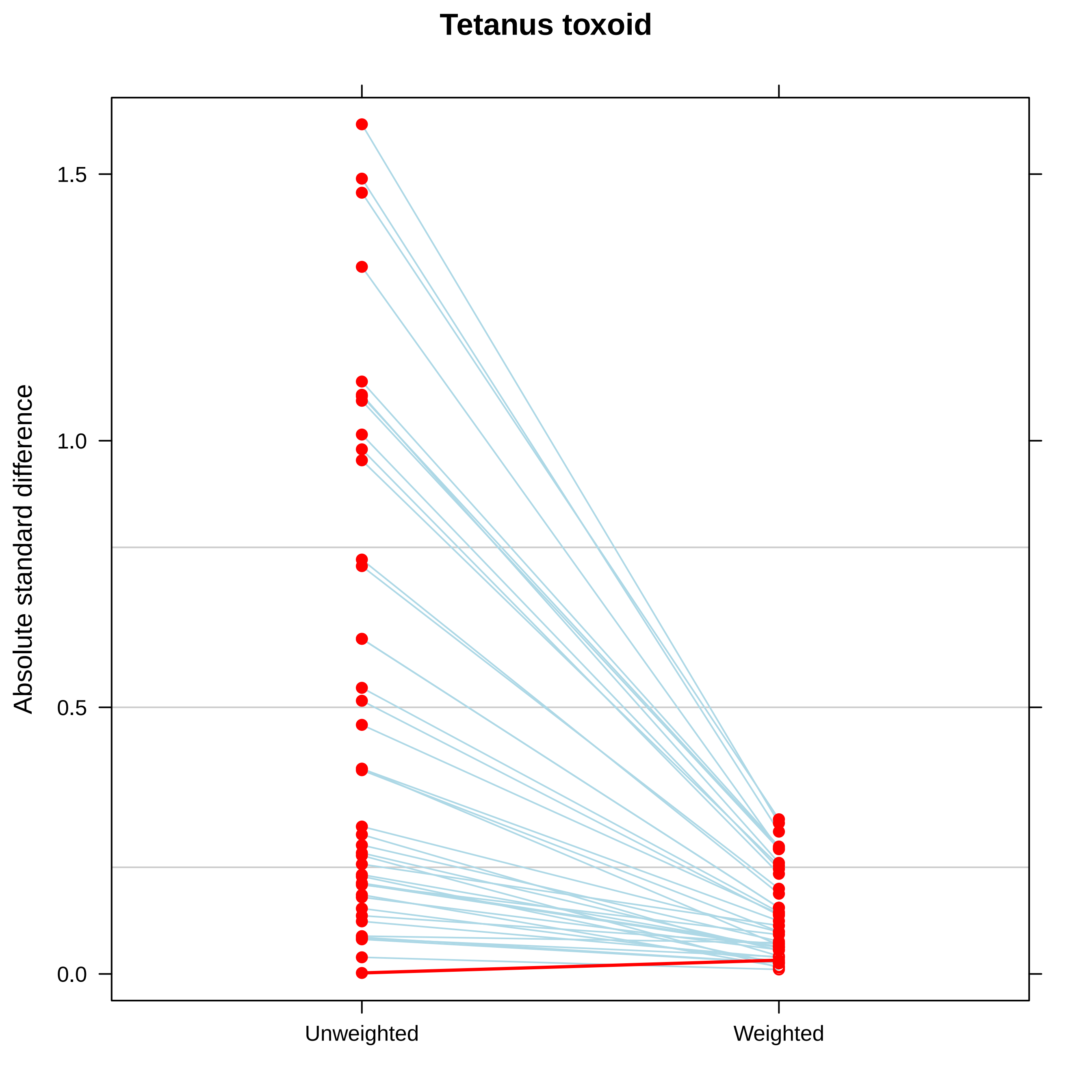

Tetanus toxoid
Absolute standard difference
1.5
1.0
0.5
0.0
Unweighted
Weighted

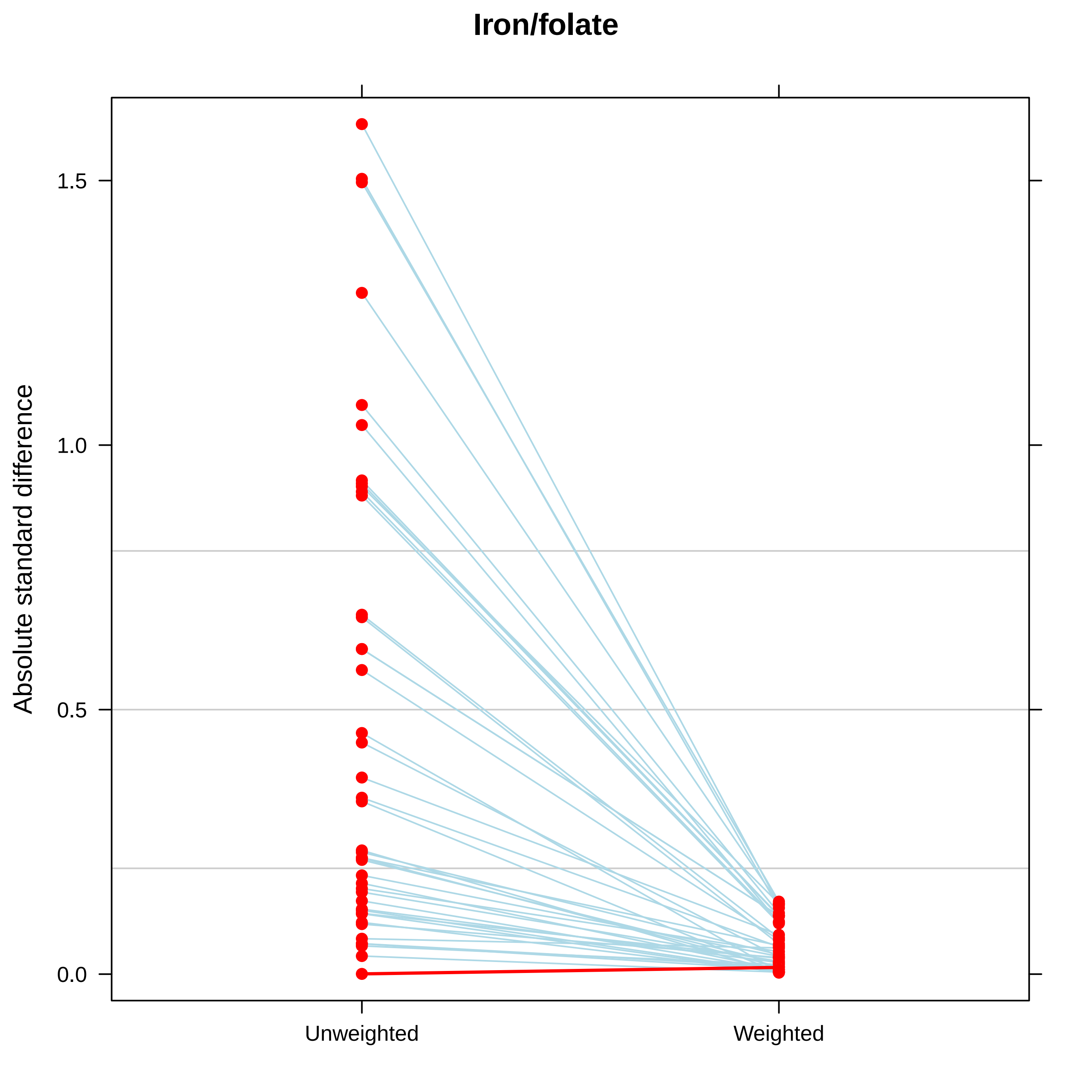
Iron/folate
Absolute standard difference
1.5
1.0
0.5
0.0
Unweighted
Weighted

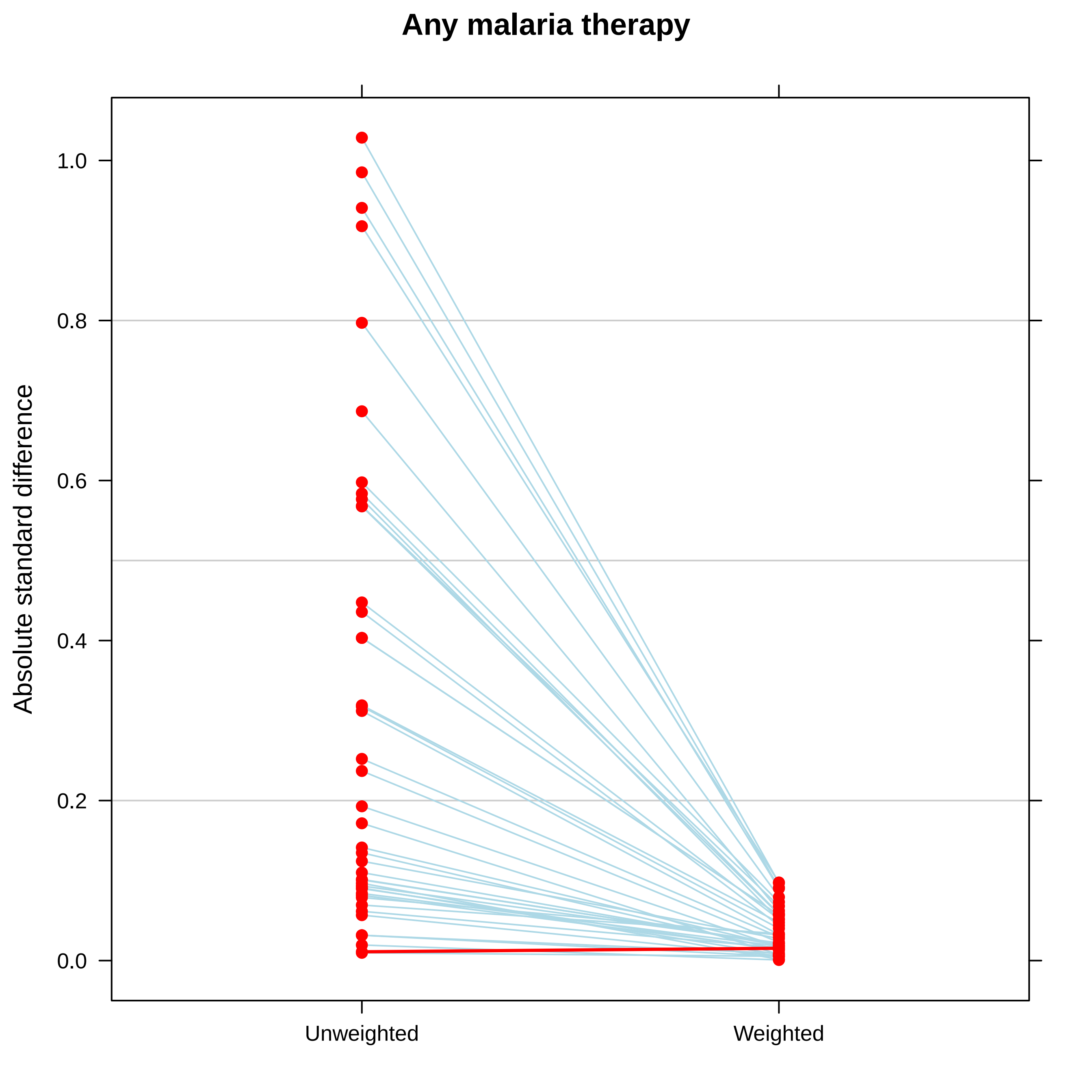
Any malaria therapy
Absolute standard difference
1.0
0.8
0.6
0.4
0.2
0.0
Unweighted
Weighted

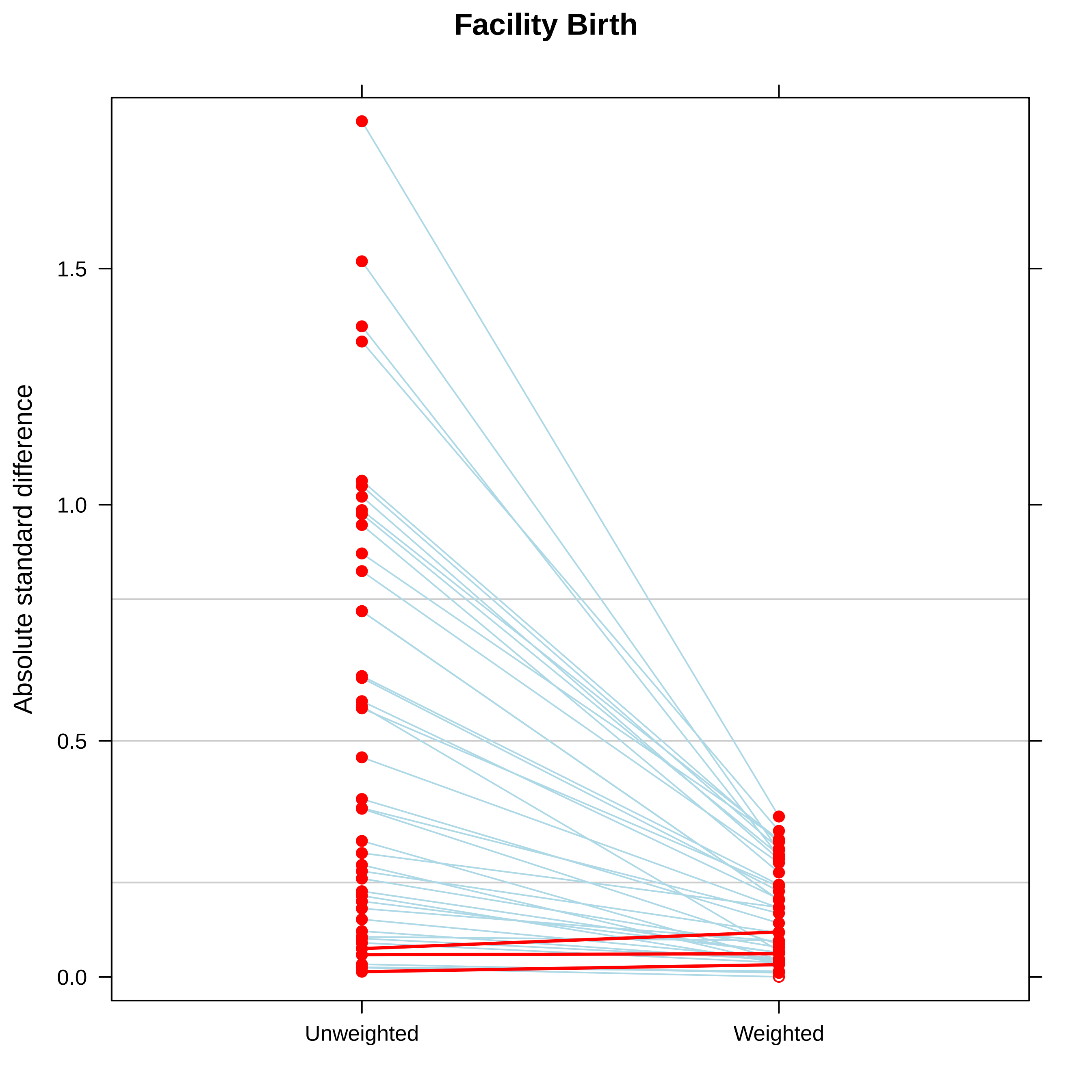
Facility Birth
Absolute standard difference
0.0
0.5
1.0
1.5
Unweighted
Weighted

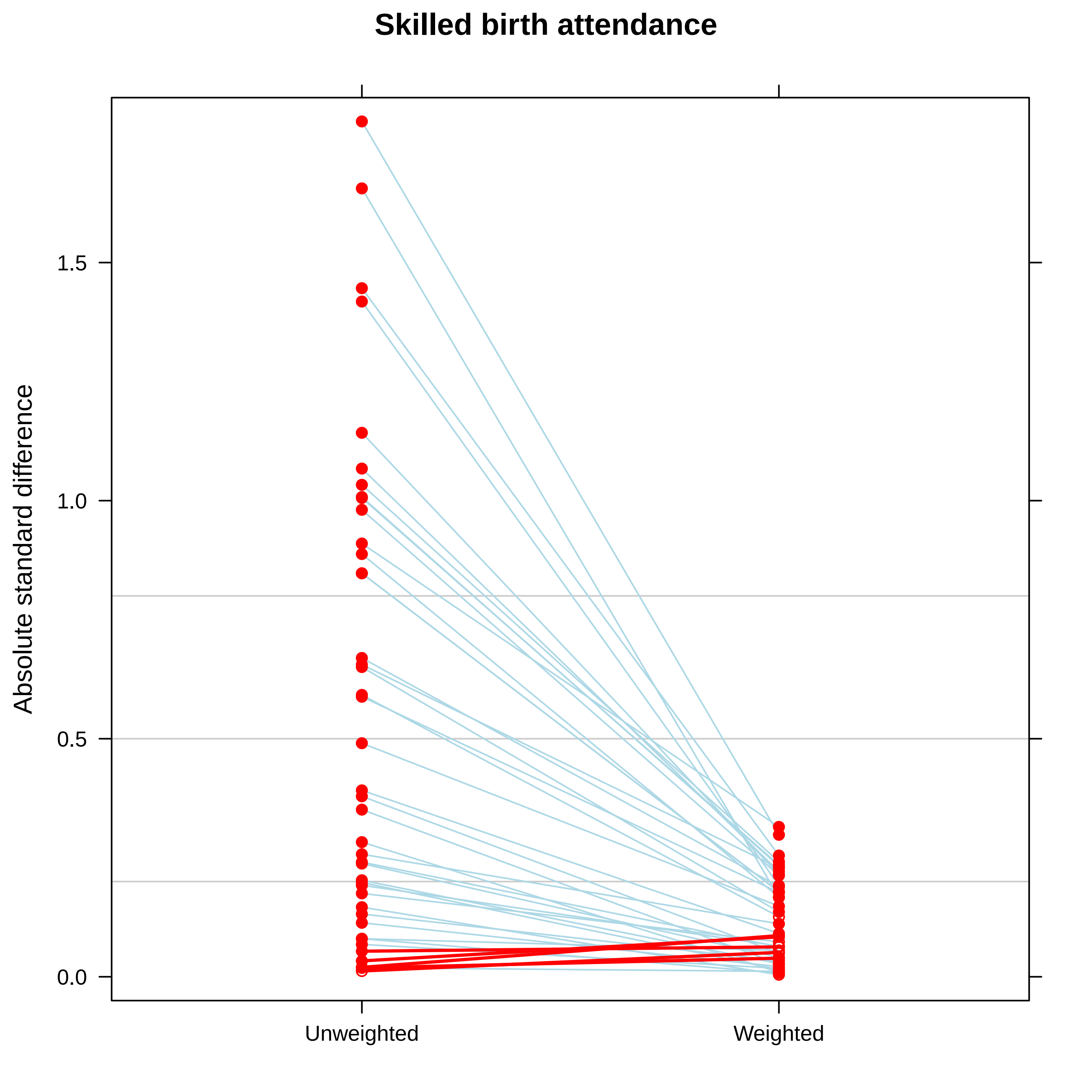

Skilled birth attendance
Absolute standard difference
1.5
1.0
0.5
0.0
Unweighted
Weighted

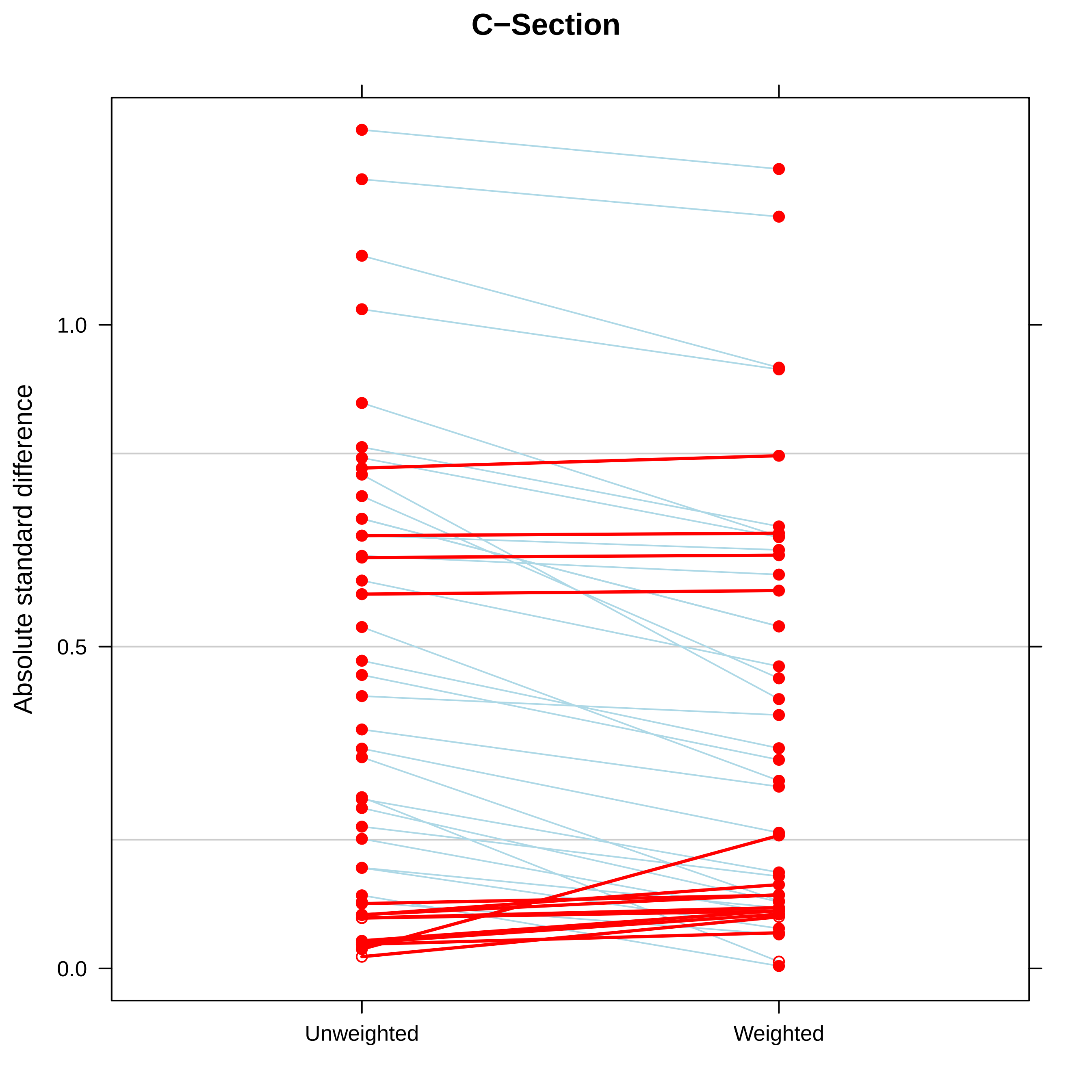
C−Section
Absolute standard difference
1.0
0.5
0.0
Unweighted
Weighted

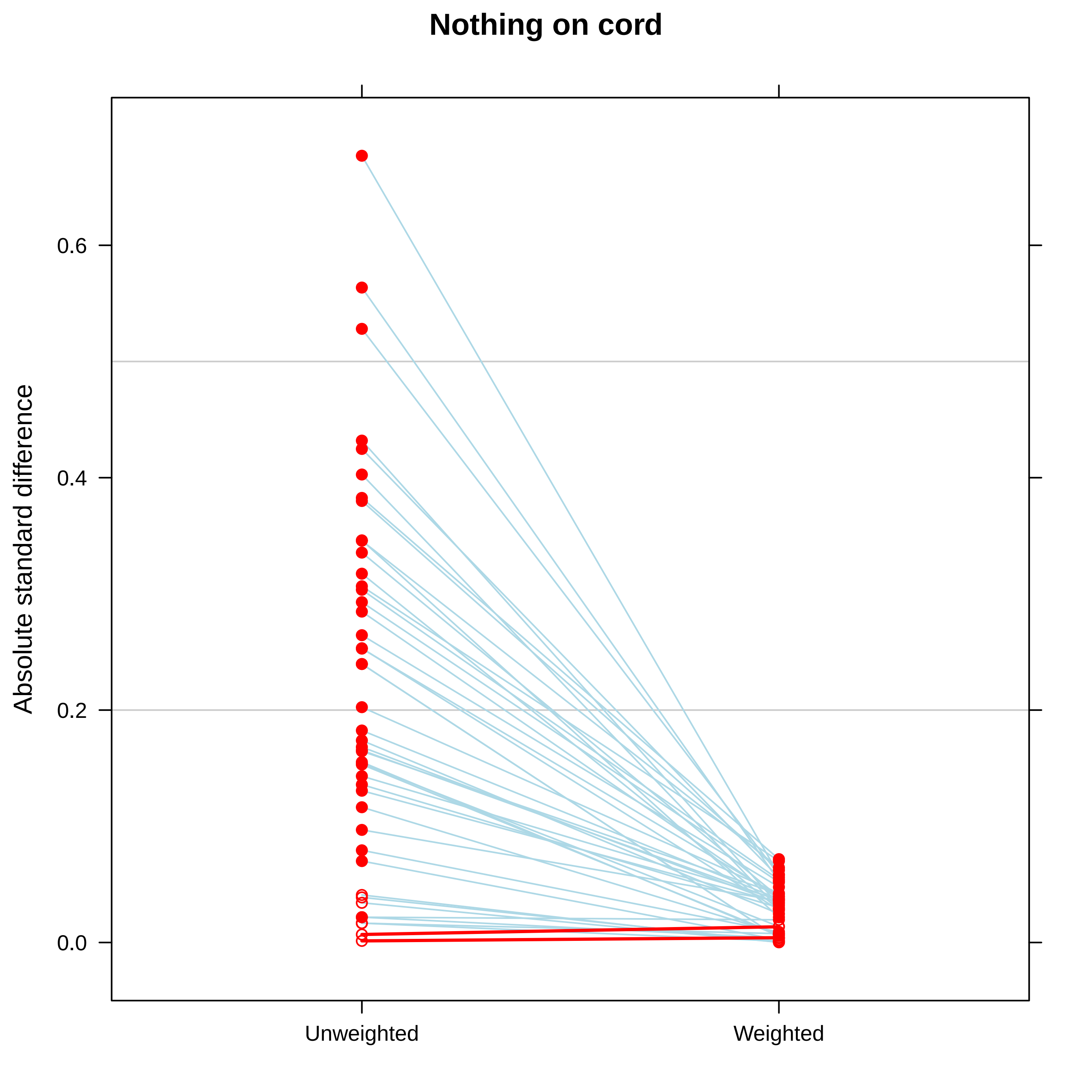
Nothing on cord
Absolute standard difference
0.0
0.2
0.4
0.6
Unweighted
Weighted

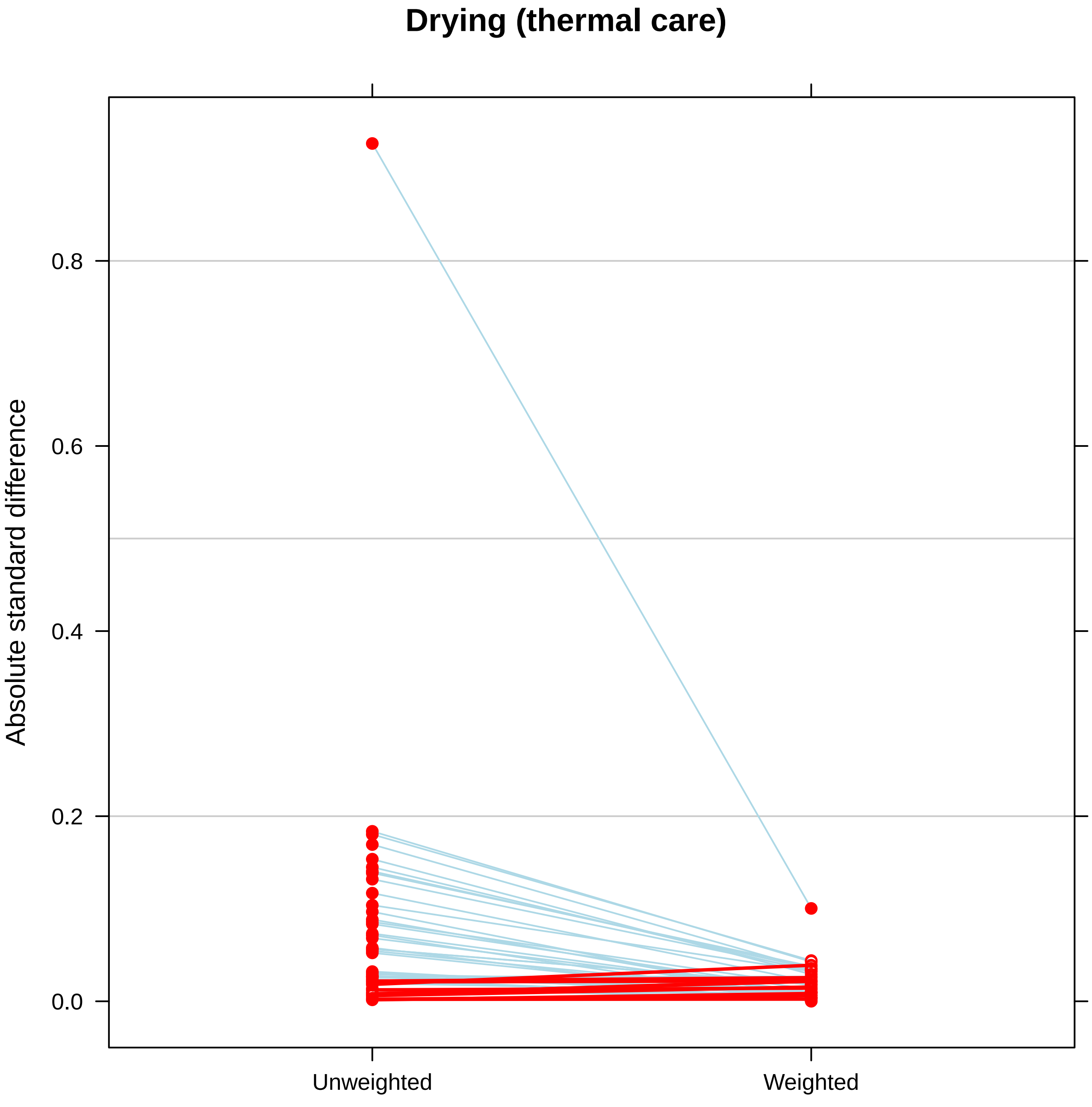

Drying (thermal care)
Absolute standard difference
0.8
0.6
0.4
0.2
0.0
Unweighted
Weighted

Skin to skin

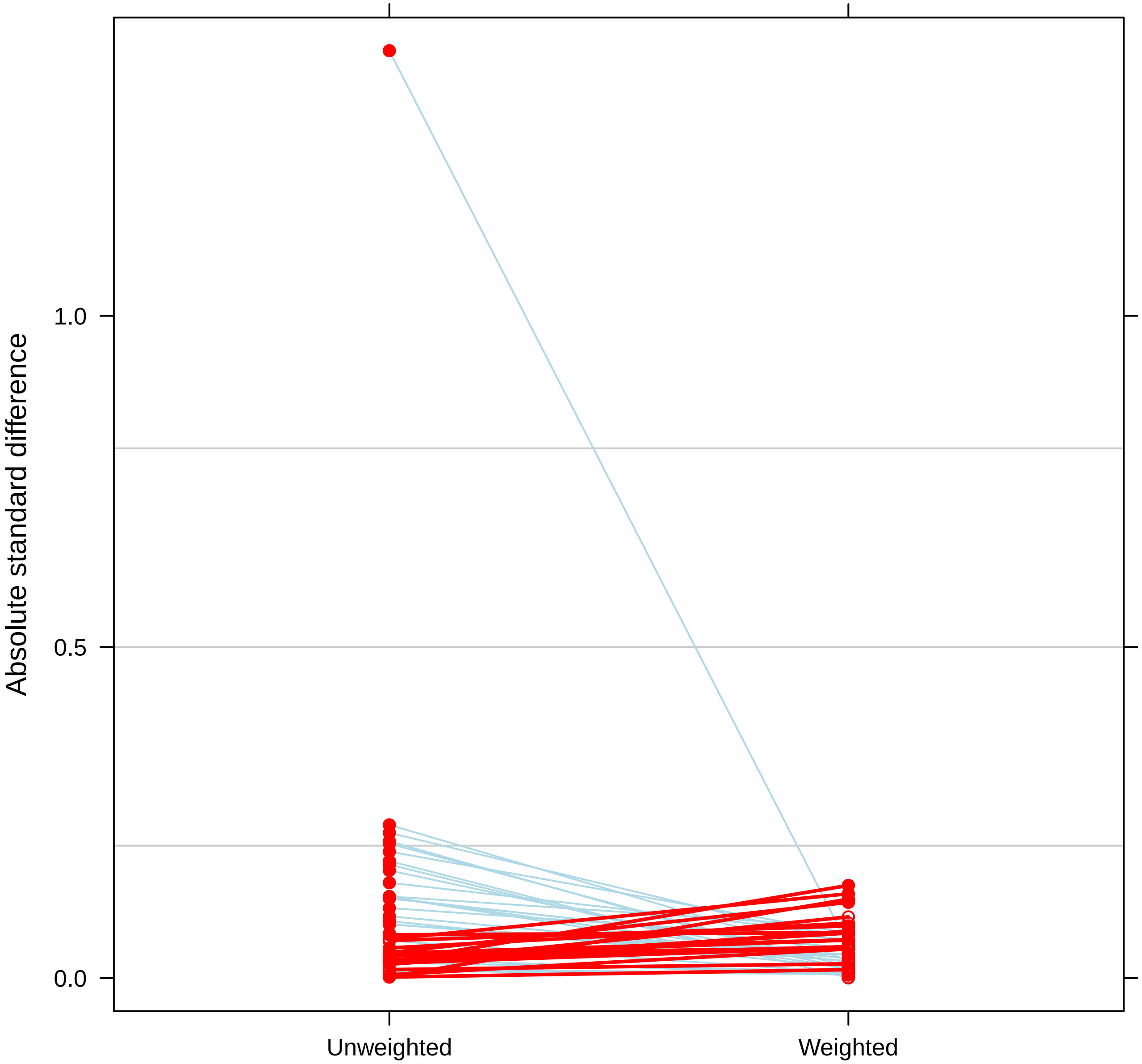

Absolute standard difference
1.0
0.5
0.0
Unweighted
Weighted

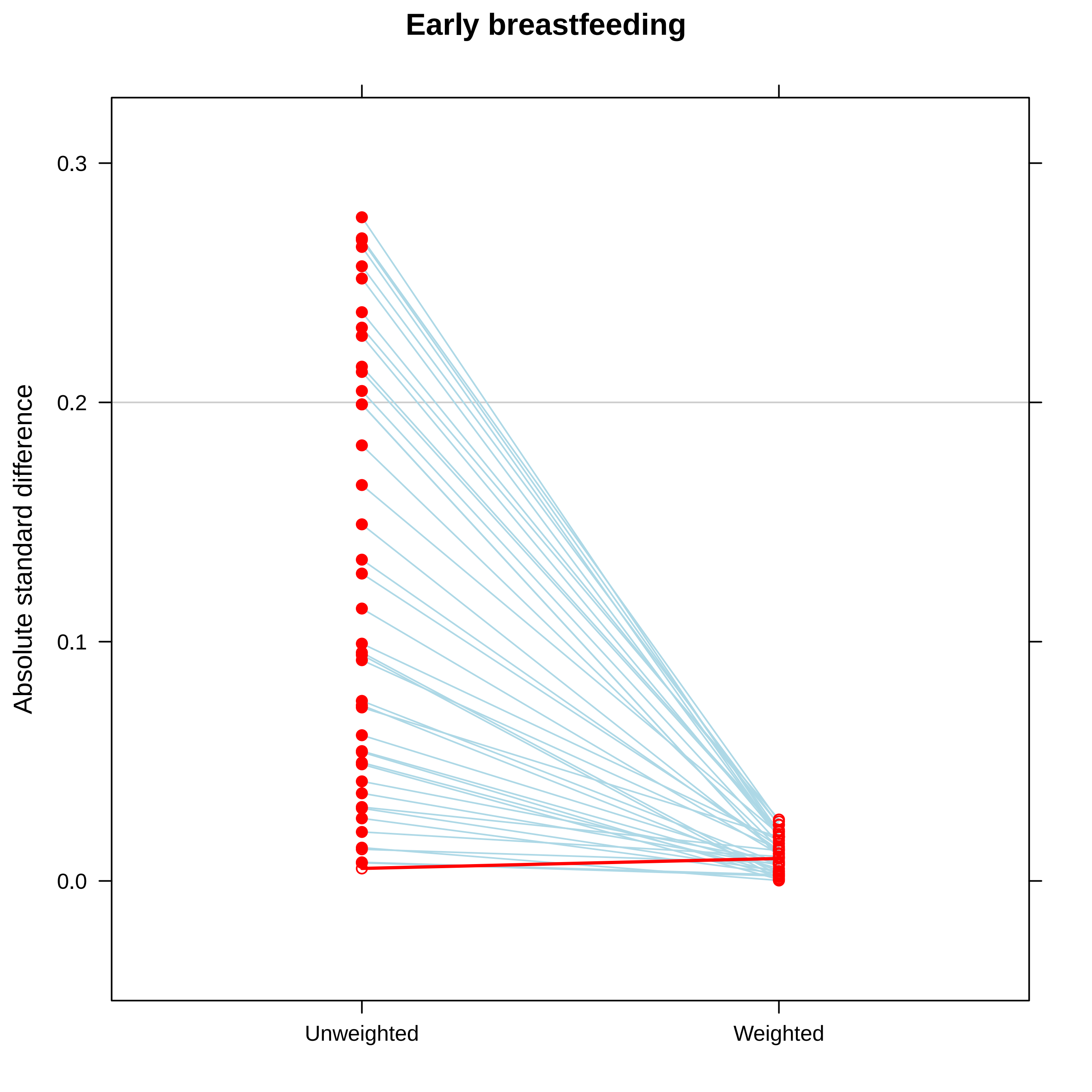

Early breastfeeding
Absolute standard difference
0.3
0.2
0.1
0.0
Unweighted
Weighted

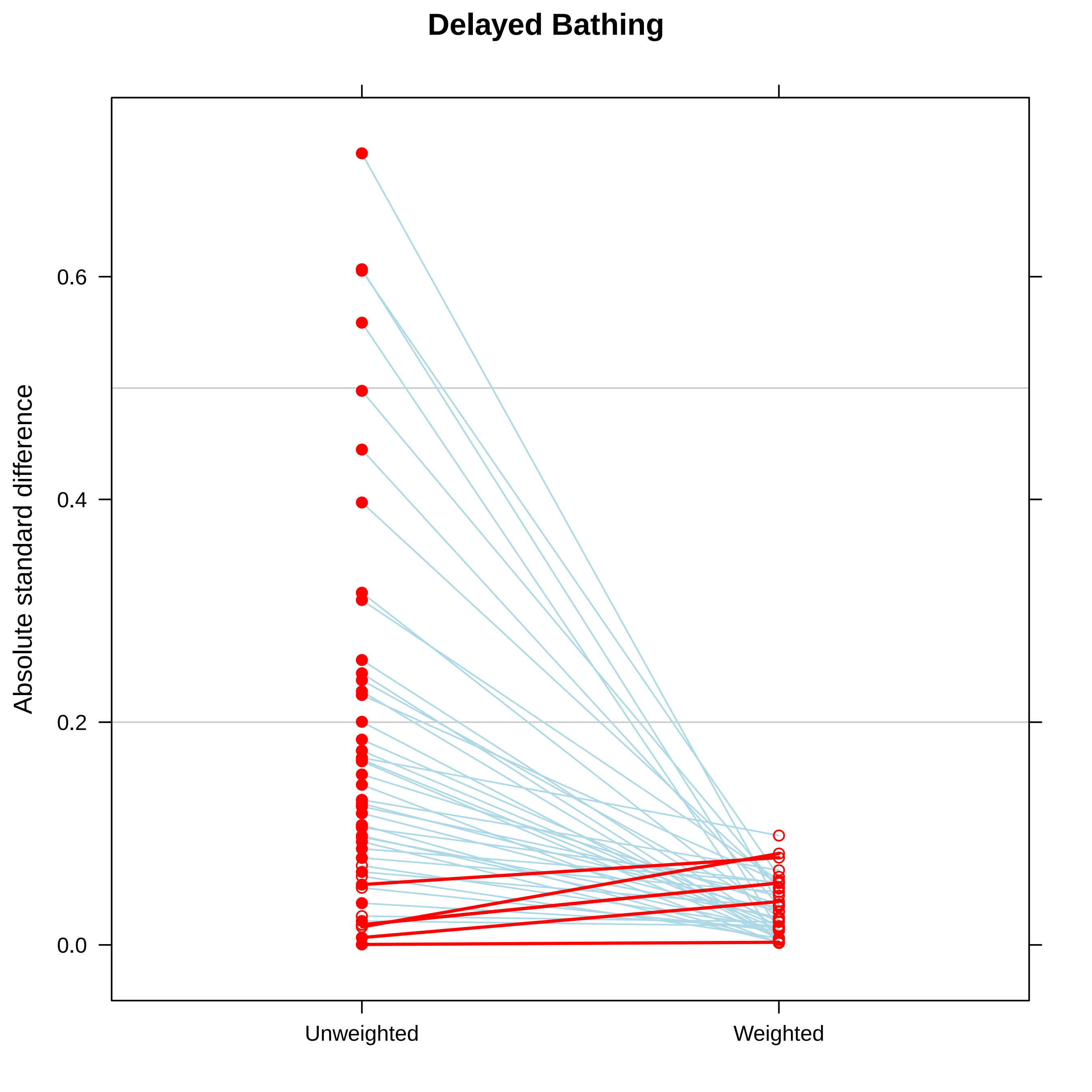
Delayed Bathing
Absolute standard difference
0.0
0.2
0.4
0.6
Unweighted
Weighted

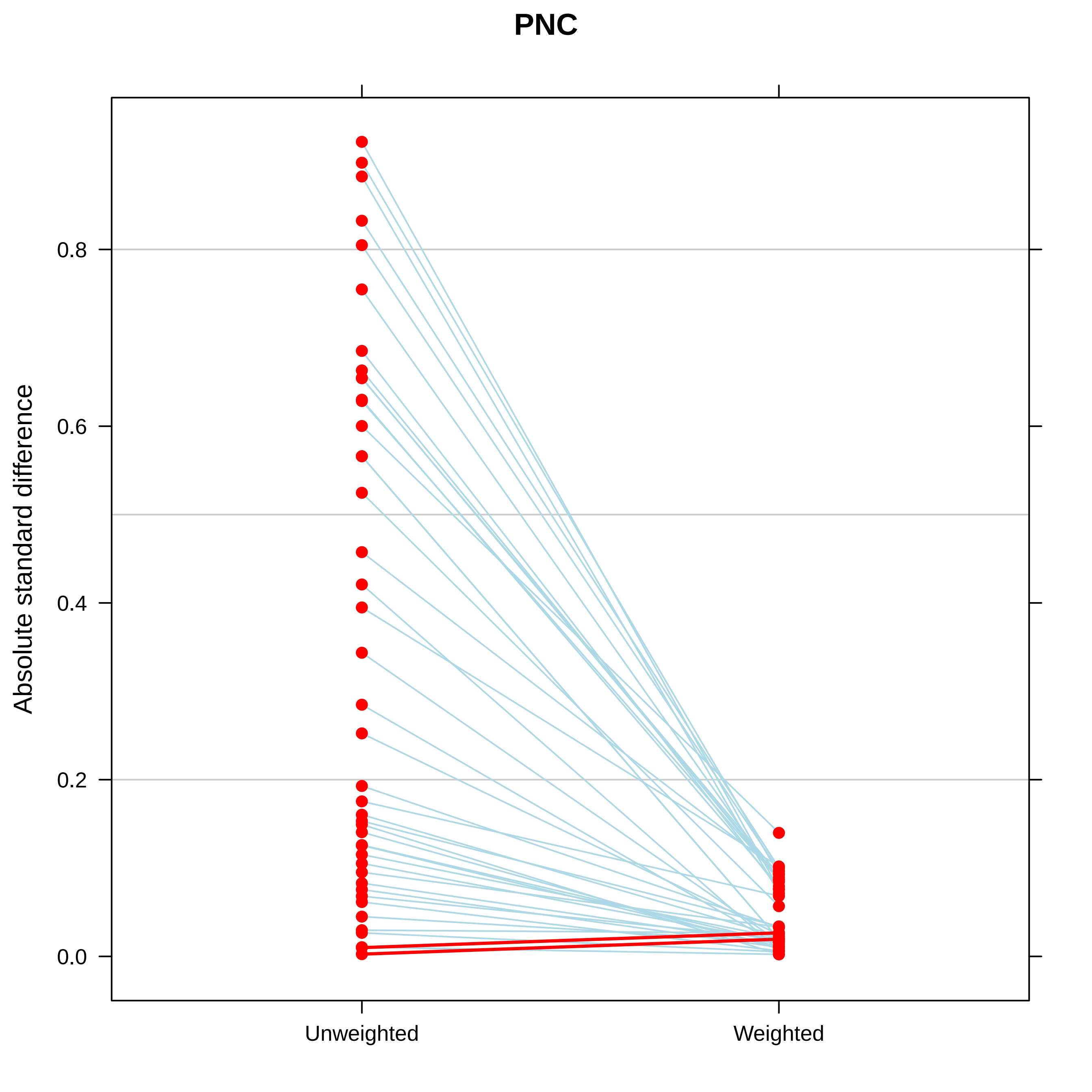
PNC
Absolute standard difference
0.0
0.2
0.4
0.6
0.8
Unweighted
Weighted
