# Supplementary material for: Using propensity scores to estimate the effectiveness of maternal and newborn interventions to reduce neonatal mortality in Nigeria
Source: BMC Pregnancy Childbirth. 2020 Sep 14;20:534. doi: 10.1186/s12884-020-03220-3 (PMC7488987; doi:10.1186/s12884-020-03220-3)
Supplement: Supplementary file 3 — Additional file 3. A summary of cause of neonatal death and the ages at neonatal death. [file 12884_2020_3220_MOESM3_ESM.docx]

**Appendix 1. Estimated Causes of neonatal mortality and age of death among neonates in the Nigeria 2013 DHS.**

**Table A1. Summary of causes of neonatal mortality among 326 deaths with physician coded verbal autopsy.**

|  | **N (unweighted)** | **Survey weighted Percent** |
| --- | --- | --- |
| ***Estimated primary underlying cause*** |  |  |
| Pneumonia | 107 | 31% |
| Birth injury/ Birth asphyxia | 61 | 23% |
| Sepsis | 44 | 13% |
| Preterm delivery | 15 | 4% |
| Meningitis | 14 | 4% |
| Diarrhea | 3 | 1% |
| Malformation | 3 | 1% |
| Tetanus | 2 | 0% |
| Other | 35 | 9% |
| Unspecified | 42 | 13% |

**Table A2. Summary of ages at death among 326 deaths with physician coded verbal autopsy.**

|  | **N (unweighted)** | **Survey weighted Percent** |
| --- | --- | --- |
| ***Reported age at death*** |  |  |
| Day of birth | 70 | 21.0% |
| 1 | 76 | 23.2% |
| 2 | 29 | 9.3% |
| 3 | 30 | 10.4% |
| 4 | 14 | 3.9% |
| 5 | 15 | 5.4% |
| 6 | 9 | 1.8% |
| 7 | 16 | 4.6% |
| 8 | 16 | 4.7% |
| 9 | 5 | 1.7% |
| 10 | 5 | 1.3% |
| 11 | 2 | 1.1% |
| 12 | 4 | 1.3% |
| 13 | 4 | 1.2% |
| 14 | 8 | 1.4% |
| 15 | 5 | 1.9% |
| 16 | 3 | 0.7% |
| 17 | 3 | 1.7% |
| 18 | 0 | 0.0% |
| 19 | 1 | 0.3% |
| 20 | 1 | 0.3% |
| 21 | 5 | 2.0% |
| 22 | 1 | 0.3% |
| 23 | 0 | 0.0% |
| 24 | 1 | 0.3% |
| 25 | 0 | 0.0% |
| 26 | 1 | 0.2% |
| 27 | 2 | 0.2% |
