# Supplementary material for: Using propensity scores to estimate the effectiveness of maternal and newborn interventions to reduce neonatal mortality in Nigeria
Source: BMC Pregnancy Childbirth. 2020 Sep 14;20:534. doi: 10.1186/s12884-020-03220-3 (PMC7488987; doi:10.1186/s12884-020-03220-3)
Supplement: Supplementary file 4 — Additional file 4. Poisson regression for the association between each of 18 interventions and all cause, IPRE, and infection related mortality. [file 12884_2020_3220_MOESM4_ESM.docx]

**Appendix 4. Crude mortality risk for 18 interventions for neonatal survival among a representative sample of households in Nigeria 2013 DHS.**

**Table A1. Summary of coverages for chosen interventions in the Nigeria survey, among decedents and survivors, and the crude relative risk of all-cause neonatal mortality in the five years prior to survey, for 19685 livebirths in the five years prior to survey in which all primary neonatal interventions (except those only measured for home deliveries) were reported.**

|  | **Overall** | | **Coverage** | | **Relative Risk** | | |
| --- | --- | --- | --- | --- | --- | --- | --- |
|  |  |  |  |  |  |  |  |
|  | **n** | **Coverage** | **Deaths** | **Survivors** | **Est** | **(95% CI)** | **p** |
| ***Structures/Resources*** |  |  |  |  |  |  |  |
| Mother is primary decision maker | 19685 | 3% | 2% | 3% | 0.84 | (0.39, 1.82) | 0.659 |
| Distance is not a problem for mother's health care | 19685 | 69% | 72% | 68% | 1.19 | (0.95, 1.47) | 0.126 |
| ***Interventions related to the antenatal period*** |  |  |  |  |  |  |  |
| ANC 1 visit | 19685 | 60% | 57% | 60% | 0.88 | (0.73, 1.07) | 0.200 |
| ANC 4 visits | 19685 | 51% | 49% | 51% | 0.90 | (0.75, 1.08) | 0.253 |
| At least one ANC intervention | 19685 | 62% | 60% | 62% | 0.90 | (0.75, 1.10) | 0.310 |
| Four ANC interventions | 19685 | 37% | 36% | 38% | 0.95 | (0.77, 1.18) | 0.661 |
| Tetanus Toxoid during pregnancy | 19685 | 59% | 58% | 59% | 0.96 | (0.80, 1.16) | 0.687 |
| Iron/folate during pregnancy | 19685 | 63% | 62% | 63% | 0.94 | (0.78, 1.13) | 0.492 |
| Any Malaria preventive therapy during pregnancy | 19685 | 48% | 50% | 48% | 1.06 | (0.87, 1.30) | 0.547 |
| ***Interventions related to labor and delivery*** |  |  |  |  |  |  |  |
| Institutional birth | 19685 | 37% | 40% | 37% | 1.11 | (0.89, 1.37) | 0.351 |
| Skilled attendant during birth | 19685 | 40% | 44% | 40% | 1.16 | (0.94, 1.43) | 0.174 |
| Delivered by C-Section | 19685 | 2% | 6% | 2% | 2.78 | (1.89, 4.08) | **< 0.001** |
| ***Interventions related to the postnatal period*** |  |  |  |  |  |  |  |
| Dry cord care (nothing on cord) ^1^ | 12157 | 64% | 71% | 64% | 1.35 | (1.02, 1.79) | **0.038** |
| Neonate dried after birth^1^ | 12157 | 28% | 28% | 28% | 1.01 | (0.76, 1.34) | 0.930 |
| Skin-to-skin contact after birth^1^ | 12157 | 9% | 8% | 9% | 0.81 | (0.53, 1.23) | 0.326 |
| Early breastfeeding (within one hour) | 19685 | 34% | 16% | 35% | 0.38 | (0.30, 0.49) | **< 0.001** |
| Delayed bathing 24 hours or more^1^ | 12157 | 4% | 4% | 4% | 1.19 | (0.56, 2.51) | 0.654 |
| PNC within 2 days of births | 19685 | 15% | 9% | 15% | 0.59 | (0.40, 0.87) | **0.007** |

^1^Only measured among home deliveries.

**Table A2. Summary of coverages for chosen interventions in the Nigeria survey, among decedents and survivors, and the crude relative risk of neonatal mortality due to sepsis, pneumonia, meningitis, diarrhea, or tetanus in the five years prior to survey, for 19685 livebirths in the five years prior to survey in which all primary neonatal interventions (except those only measured for home deliveries) were reported.**

|  | **Overall** | | **Coverage** | | **Crude** | | |
| --- | --- | --- | --- | --- | --- | --- | --- |
|  |  |  |  |  | **Relative Mortality Risk** | | |
|  | **n** | **Coverage** | **Deaths due to infections**^2^  **(n=171)** | **Survivors** | **Est** | **95% CI** | **p** |
| ***Structures/Resources*** |  |  |  |  |  |  |  |
| Mother is primary decision maker | 19685 | 3% | 1% | 3% | 0.30 | (0.07, 1.22) | 0.093 |
| Distance is not a problem for mother's health care | 19685 | 69% | 74% | 69% | 1.29 | (0.87, 1.92) | 0.201 |
| ***Interventions related to the antenatal period*** |  |  |  |  |  |  |  |
| ANC 1 visit | 19685 | 60% | 58% | 60% | 0.90 | (0.63, 1.28) | 0.558 |
| ANC 4 visits | 19685 | 51% | 46% | 51% | 0.81 | (0.58, 1.14) | 0.231 |
| At least one ANC intervention | 19685 | 62% | 60% | 62% | 0.89 | (0.63, 1.26) | 0.503 |
| Four ANC interventions | 19685 | 37% | 38% | 37% | 1.01 | (0.68, 1.51) | 0.946 |
| Tetanus toxoid during pregnancy | 19685 | 59% | 59% | 59% | 0.98 | (0.69, 1.39) | 0.902 |
| Iron/folate during pregnancy | 19685 | 63% | 60% | 63% | 0.87 | (0.61, 1.25) | 0.458 |
| Any malaria preventive therapy during pregnancy | 19685 | 48% | 48% | 48% | 0.97 | (0.67, 1.41) | 0.879 |
| ***Interventions related to labor and delivery*** |  |  |  |  |  |  |  |
| Institutional birth | 19685 | 37% | 32% | 37% | 0.78 | (0.55, 1.12) | 0.180 |
| Skilled attendant during birth | 19685 | 40% | 35% | 40% | 0.82 | (0.57, 1.16) | 0.258 |
| Delivered by C-Section | 19685 | 2% | 6% | 2% | 2.49 | (1.06, 5.68) | **0.030** |
| ***Interventions related to the postnatal period*** |  |  |  |  |  |  |  |
| Dry cord care (nothing on cord) ^1^ | 12157 | 64% | 65% | 64% | 1.06 | (0.66, 1.70) | 0.816 |
| Neonate dried after birth^1^ | 12157 | 28% | 32% | 28% | 1.17 | (0.73, 1.90) | 0.512 |
| Skin-to-skin contact after birth^1^ | 12157 | 9% | 6% | 9% | 0.68 | (0.29, 1.61) | 0.377 |
| Early breastfeeding (within one hour) | 19685 | 34% | 22% | 34% | 0.53 | (0.35, 0.81) | **0.003** |
| Delayed bathing 24 hours or more^1^ | 12157 | 4% | 5% | 4% | 1.39 | (0.52, 3.77) | 0.513 |
| Postnatal health contact within 2 days of birth | 19685 | 15% | 10% | 15% | 0.62 | (0.35, 1.11) | 0.110 |

^1^Only measured for home deliveries.

^2^Caused by diarrhea, sepsis, meningitis, pneumonia, or tetanus, where cause of death was determined by expert algorithm of verbal autopsy responses.

**Table A3. Summary of coverages for chosen interventions in the Nigeria survey, among decedents and survivors, and the crude relative risk of neonatal mortality due to birth injury or birth asphyxia in the five years prior to survey, for 19685 livebirths in the five years prior to survey in which all primary neonatal interventions (except those only measured for home deliveries) were reported.**

|  | **Overall** | | **Coverage** | | **Crude** | | |
| --- | --- | --- | --- | --- | --- | --- | --- |
|  |  |  |  |  | **Relative Mortality Risk** | | |
|  | **n** | **Coverage** | **Deaths due to birth asphyxia**  **(n=74)** | **Survivors** | **Est** | **95% CI** | **p** |
| ***Structures/Resources*** |  |  |  |  |  |  |  |
| Mother is primary decision maker | 19685 | 3% | 3% | 3% | 1.18 | (0.32 ,4.28) | 0.806 |
| Distance is not a problem for mother's health care | 19685 | 69% | 73% | 69% | 1.27 | (0.70 ,2.30) | 0.439 |
| ***Interventions related to the antenatal period*** |  |  |  |  |  |  |  |
| ANC 1 visit | 19685 | 60% | 57% | 60% | 0.87 | (0.51 ,1.48) | 0.598 |
| ANC 4 visits | 19685 | 51% | 49% | 51% | 0.92 | (0.56 ,1.52) | 0.750 |
| At least one ANC intervention | 19685 | 62% | 63% | 62% | 1.04 | (0.62 ,1.76) | 0.880 |
| Four ANC interventions | 19685 | 37% | 32% | 38% | 0.78 | (0.45 ,1.36) | 0.386 |
| Tetanus toxoid during pregnancy | 19685 | 59% | 55% | 59% | 0.85 | (0.51 ,1.43) | 0.542 |
| Iron/folate during pregnancy | 19685 | 63% | 61% | 63% | 0.91 | (0.53 ,1.54) | 0.717 |
| Any malaria preventive therapy during pregnancy | 19685 | 48% | 43% | 48% | 0.82 | (0.48 ,1.39) | 0.463 |
| ***Interventions related to labor and delivery*** |  |  |  |  |  |  |  |
| Institutional birth | 19685 | 37% | 47% | 37% | 1.48 | (0.87 ,2.53) | 0.150 |
| Skilled attendant during birth | 19685 | 40% | 55% | 40% | 1.84 | (1.09 ,3.11) | **0.023** |
| Delivered by C-Section | 19685 | 2% | 6% | 2% | 3.65 | (1.21, 11.02) | **0.022** |
| ***Interventions related to the postnatal period*** |  |  |  |  |  |  |  |
| Dry cord care (nothing on cord) ^1^ | 12157 | 64% | 70% | 64% | 1.34 | (0.69 ,2.58) | 0.388 |
| Neonate dried after birth^1^ | 12157 | 28% | 18% | 28% | 0.55 | (0.23 ,1.31) | 0.176 |
| Skin-to-skin contact after birth^1^ | 12157 | 9% | 4% | 9% | 0.44 | (0.11 ,1.71) | 0.236 |
| Early breastfeeding (within one hour) | 19685 | 34% | 10% | 34% | 0.21 | (0.09 ,0.47) | **< 0.001** |
| Delayed bathing 24 hours or more^1^ | 12157 | 4% | 0% | 4% | 0.00 | (0.00 ,0.01) | **< 0.001** |
| Postnatal health contact within 2 days of birth | 19685 | 15% | 9% | 15% | 0.56 | (0.24 ,1.27) | 0.164 |

^1^Only measured for home deliveries.
