# Supplementary material for: Using propensity scores to estimate the effectiveness of maternal and newborn interventions to reduce neonatal mortality in Nigeria
Source: BMC Pregnancy Childbirth. 2020 Sep 14;20:534. doi: 10.1186/s12884-020-03220-3 (PMC7488987; doi:10.1186/s12884-020-03220-3)
Supplement: Supplementary file 5 — Additional file 5. A table summarizing the considerations of analysis at all stages. [file 12884_2020_3220_MOESM5_ESM.docx]

**Analysis considerations**

| **Phase** | **Consideration** |
| --- | --- |
| Choosing Interventions and resources for analysis | Documented in household survey |
|  | Expected to be causally related to neonatal survival from prior research |
|  | Can be balanced for confounding factors with a propensity score |
| Choosing potential confounding factors | Documented in household survey |
|  | Expected to be related to both neonatal survival and whether a structure/resource or intervention was available. |
|  | Expected to be measured well in household surveys. |
| Choosing analysis methods | Robust to confounding |
|  | Flexibility for adjusting for many potential confounders |
|  | Accommodating survey design |
| Diagnostics for chosen analysis | Checking balance of confounding factors |
